# Activation of Cell-Intrinsic Signaling in CAR-T Cells via a Chimeric IL7R Domain

**DOI:** 10.1158/2767-9764.CRC-24-0286

**Published:** 2024-09-09

**Authors:** Stamatia C. Vorri, Natalie J. Holl, Michael Leeming, Petya Apostolova, Andrew Marple, Jonas W. Ravich, Ata Canbaz, Ruyan Rahnama, Jun Choe, Arjun Modi, Adam D. Fearnow, Scott T.R. Walsh, Erika L. Pearce, Ravi Varadhan, Challice L. Bonifant

**Affiliations:** 1 Department of Oncology, The Sidney Kimmel Comprehensive Cancer Center, Johns Hopkins University School of Medicine, Baltimore, Maryland.; 2 Bio21 Molecular Science and Biotechnology Institute, The University of Melbourne, Victoria, Australia.; 3 Bloomberg-Kimmel Institute for Cancer Immunotherapy, Johns Hopkins University School of Medicine, Baltimore, Maryland.; 4 Division of Hematology, University Hospital Basel, Basel, Switzerland.; 5 Department of Pediatrics, Johns Hopkins University School of Medicine, Baltimore, Maryland.; 6 Department of Pathology, Johns Hopkins University School of Medicine, Baltimore, Maryland.; 7 Chemical Biology Laboratory, National Cancer Institute, National Institutes of Health, Frederick, Maryland.

## Abstract

Chimeric antigen receptor (CAR) T cells can effectively treat leukemias, but sustained antitumor responses can be hindered by a lack of CAR T-cell persistence. Cytotoxic effector T cells are short-lived, and establishment of CAR-T cells with memory to ensure immune surveillance is important. Memory T cells depend on cytokine support, with IL7 activation of the IL7 receptor (IL7R) being critical. However, IL7R surface expression is negatively regulated by exposure to IL7. We aimed to support CAR T-cell persistence by equipping CAR-T cells with a sustained IL7Rα signal. We engineered T cells to constitutively secrete IL7 or to express an anti–acute myeloid leukemia–targeted IL7Rα–chimeric cytokine receptor (CCR) and characterized the phenotype of these cell types. Canonical downstream signaling was activated in CCR-T cells with IL7R activation. When coexpressed with a cytotoxic CAR, functionality of both the CCR and CAR was maintained. We designed hybrid CAR–CCR and noted membrane proximity of the intracellular domains as vital for signaling. These data show cell-intrinsic cytokine support with canonical signaling, and functionality can be provided via expression of an IL7Rα domain whether independently expressed or incorporated into a cytotoxic CAR for use in anticancer therapy.

**Significance:**

To improve the phenotype of tumor-directed T-cell therapy, we show that provision of cell-intrinsic IL7R-mediated signaling is preferable to activation of cells with exogenous IL7. We engineer this signaling via independent receptor engineering and incorporation into a CAR and validate maintained antigen-specific cytotoxic activity.

## Introduction

Chimeric antigen receptor (CAR) T cells have shown important clinical efficacy against leukemia, and their use can lead to durable disease remission (1–6). However, the antileukemia efficacy of CAR-T cells may be short-lived without CAR T-cell persistence (1, 7, 8). It is hypothesized that lack of persistence correlates with enrichment of activated effector T cells in CAR-T products. Although effector T cells are highly proliferative in response to a specific antigen stimulus, they are short-lived. When the antigen stimulation is no longer present, this T-cell population contracts, leaving memory counterparts as a pool of long-lived T cells that conduct immune surveillance and can rapidly re-expand upon antigen re-encounter (9). For patients treated with CAR-T cells, long-lived antitumor disease surveillance is likely needed to guard against relapse. Supporting this hypothesis, evaluations of CAR T-cell product characteristics have identified phenotypic correlates of disease-free survival, including enrichment of CAR-T subsets with naïve and central memory programming (8, 10).

T cells have dynamic metabolic potential. Naïve, effector, and memory T cells have distinct metabolic profiles suited for their precise immunologic roles. Naïve and memory T cells primarily use oxidative phosphorylation to meet their energy needs and transition to rely on aerobic glycolysis as they differentiate into actively proliferating effectors. However, memory T cells have increased mitochondrial mass and higher maximum respiratory capacity when compared with naïve and effector T cells (11). These traits prime memory T cells to quickly meet the metabolic demand of re-expansion when needed. Infusion of CAR T-cell products with a higher proportion of these memory-like subsets has correlated with superior disease-free survival (12, 13) while possibly decreasing the risk of cytokine release syndrome (14). Memory T cells rely on IL7 receptor (IL7R) stimulation for homeostatic survival (15–17). Notably, in the lymphopenic host, IL7 is a critical cytokine that contributes to restoring the memory T-cell reservoir (18). IL7 activates a heterodimeric receptor, comprised of the IL7Rα chain (CD127) and the common γ chain (γ_c_; CD132; ref. 19). IL7 first binds IL7Rα, which then dimerizes with γ_c_ and transduces intracellular signaling via JAK1 and JAK3 heterophosphorylation, followed by PI3K and STAT5 activation.

The expression of IL7Rα regulates IL7 signaling rather than cytokine abundance (20). IL7Rα is highly expressed on naïve T cells yet is downregulated in effector T cells. Memory T cells retain IL7Rα expression and require IL7 for survival. Overexpression of IL7 increases memory T-cell numbers (21), and supplemental IL7 has therefore been investigated as an adjuvant for adoptive T-cell therapies (22). However, IL7 stimulation leads to IL7Rα downregulation as a negative feedback mechanism (23). Additionally, systemic IL7 administration can have toxic side effects due to unspecific activation of the IL7R in T and other immune cells (24). Notably, the IL7 half-life in humans is as short as 5 hours after intravenous administration (25) and 12.5 hours after subcutaneous (26) administration. This combination of potential toxicity and need for frequent injections complicates the use of IL7 administration as support for adoptive cell therapy. Cytokine bioavailability may be enhanced with pharmacologic modification (27, 28); however, phase I studies of a long-acting IL7 conjugate noted development of anti–conjugate-directed antibodies in 100% of healthy participants after injection (29). The use of IL7 as therapy is therefore challenged by unfavorable pharmacokinetics, potential toxicity, and immunogenicity.

T cells can be engineered to constitutively secrete IL7 to stimulate engineered and neighboring cells (30–34). Due to rapid IL7Rα downregulation, (35) secreted IL7 may not support T cells in the long term and has the potential for locally increasing the cytokine to toxic levels. For these reasons, we sought alternative strategies to activate signaling downstream of the IL7R in T cells. We (36) and others (37) have utilized IL7Rα expression in the context of engineered T cells as a mechanism to support T-cell survival, that is, static, cytokine-independent, and not subject to endogenous IL7Rα expression. We have previously used a chimeric cytokine receptor (CCR), comprised of a single-chain variable fragment [scFv, 26292 (38)] that binds the leukemia-associated antigen CD123 and an IL7Rα intracellular domain (36). In this study, we compared the functionality of T cells engineered with IL7Rα signaling transmitted by (i) constitutive IL7 secretion (sIL7), (ii) expression of a constitutively active but also antigen-responsive CCR, (iii) expression of a CCR together with a cytotoxic second-generation CD28.ζ CAR, and (iv) in hybrid CCR–CAR constructs. We show that IL7Rα domain containing CCR expression supports T-cell expansion via activation of canonical IL7R signaling. With CAR and CCR coexpression, we observed maintained CAR and CCR functionality *in vitro* and *in vivo*. Finally, incorporation of the IL7Rα endodomain within the CD28.ζ CAR construct highlighted intracellular domain membrane proximity as influencing engineered receptor functionality. Inclusion of IL7Rα signaling in chimeric constructs may serve as a strategy useful in clinical development of CAR T-cell products.

## Materials and Methods

### Study design

Our study objective was to compare intrinsic with extrinsic IL7R activation of *ex vivo* activated and expanded T cells, in order to then determine the optimal strategy to achieve a desired phenotype. For all included experiments, number of replicates, number of unique T-cell donors, statistical tests, numbers of repetitions, and *P* values are reported in figure legends. Detailed numbers of replicates refer to biological replicates. All *in vitro* experiments were performed at least three times, without exclusion of data. Mice without injection of detectable labeled cells or with evident accumulation of cells in the tail only (both technically inadequate injections) were excluded from analysis to prevent confounding experimental bias. In mouse models, mice were randomly assigned to treatment groups. Experimenters injecting mice were not blinded. *In vivo* experimental endpoints were predetermined, and mice not meeting these were sacrificed at humane endpoints: paralysis, weight loss > 20%, or evident suffering.

### Cell lines

The K-562 and MV-4-11 (myelomonocytic leukemia) cell lines were purchased from the ATCC and cultured in Iscove’s modified Dulbecco’s medium (Thermo Fisher Scientific) supplemented with 10% FBS (HyClone). The Molm-13 cell line was purchased from the Leibniz Institute (DSMZ, German Collection of Microoganisms and Cell Cultures) and cultured in RPMI media supplemented with 10% FBS. CD123-expressing K-562 cells (K562.CD123) were created as described previously (39, 40). All cells used for bioluminescence imaging (BLI)–based cytotoxicity assays and our xenograft models were stably transduced with a retroviral vector carrying an enhanced GFP (eGFP) firefly luciferase fusion gene (eGFP.ffLuc; ref. 41). Cells used for long-term cytotoxicity assays were engineered to express nuclear localized eGFP. These nuclear localized sequence (NLS)-eGFP–tagged cell lines (NLS.eGFP.MV-4-11 and NLS.eGFP.Molm-13) were made by subcloning the NLS.eGFP genetic element from Addgene vector #104061 (42) into the pSFG vector backbone. The RD114-pseudotyped vector was produced as previously described (39) and used for stable transduction of target cells. GFP-positive cells were detected by flow cytometry, sorted, and maintained in the appropriate culture medium. If indicated, luciferase expression was confirmed using D-luciferin (Thermo Fisher Scientific) and subjected to quantification of bioluminescence using a BMG CLARIOstar microplate reader. All cells were cultured in a humidified atmosphere containing 5% CO_2_ at 37°C. Cell authentication (Johns Hopkins Genetic Resources Core Facility) and confirmation of *Mycoplasma*-free status (MycoAlert Mycoplasma Detection Kit, Lonza Bioscience) were carried out whenever derivative lines were produced and/or if a new stock line was expanded.

### Synthetic construct construction

**Figure 1:**
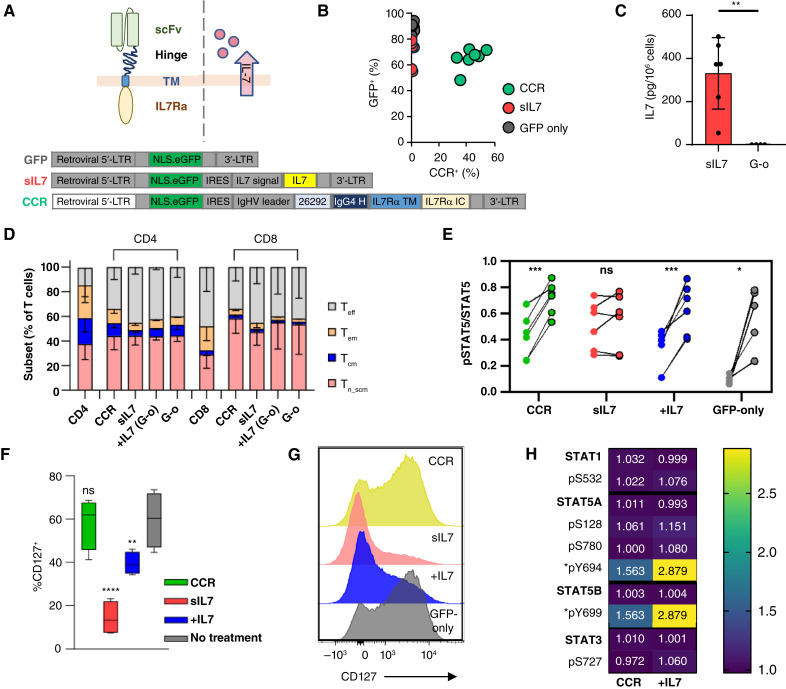
Human primary T cells can be engineered for stable IL7 pathway activation. **A,** Schematic of CCR and sIL7, as well as transgenes engineered into cells. **B,** Transduction efficiency of T cells engineered to express GFP only, IL7R + GFP, or to secrete IL7 + GFP was measured by detecting eGFP positivity and CCR expression with flow cytometry. **C,** Concentration of IL7 measured in the supernatant of sIL7-transduced T cells and control cells (G-o) in cytokine-free culture medium. **D,** Immunophenotype characterization of T-cell subsets present in healthy donor and transduced T-cell preactivation (CD4 and CD8) and on day 8 of T-cell expansion [CCR and sIL7 treated with IL7: +IL7 (G-o); G-o]. **E,** Ratio of STAT5 phosphorylation to total STAT5 expression measured with TR-FRET in indicated cell types following 24 hours of cytokine starvation and following stimulation with IL7 for 30 minutes. Comparison between untreated and IL7-treated cells. **F,** Percent cells positive for CD127 expression measured by flow cytometry. Statistical comparison is between cell type and control (G-o, no treatment). **G,** Representative histograms from flow cytometric measurement of %CD127^+^ cells of each type. Data representative of *n* = 3 to 5 independent donors. *, *P* < 0.05; **, *P* < 0.01; ***, *P* < 0.001. For DG, +IL7 indicates cells activated and expanded in the presence of IL7 prior to indicated analysis. **H,** MS proteomics analysis of IL7R signaling pathway downstream molecules for total protein and phosphosite abundance. Raw detected protein and phosphoprotein abundance were normalized to the control condition per donor and expressed as mean log_2_ fold change of three donors. *Note STAT5A pY694 and STAT5B pY699 are indistinguishable due to sequence identity. G-o, GFP-only.

CAR and CCR transgenes were designed and synthesized (GeneArt, Thermo Fisher Scientific) or subcloned from existing plasmids (36, 39). Relevant DNA sequences are included in Supplementary Table S1. These were subcloned in a pSFG retroviral expression vector (Fig. 1A) using the In-Fusion Snap Assembly kit according to the manufacturer’s instructions (Takara Bio). All sequences were validated by Sanger sequencing by the Johns Hopkins Genetic Resources Core Facility.

### *In vitro* T-cell expansion and engineering

Healthy donor peripheral blood mononuclear cells were isolated from leukopaks (Anne Arundel Medical Blood Donor Center) using density gradient centrifugation. T cells were activated with plate-bound immobilized anti-CD3ε (OKT3, Miltenyi Biotec) and anti–human CD28 (Thermo Fisher Scientific) antibodies in RPMI medium completed with 10% FBS and 2 mmol/L GlutaMAX (Thermo Fisher Scientific). Cells were maintained with supplementation of recombinant human (rh) IL2 at 200 IU/mL (Biological Resources Branch Preclinical Biorepository, NCI). For T-cell conditions supplemented with IL7, 10 ng/mL of rhIL7 (Biological Resources Branch Preclinical Biorepository, NCI) was used. When desired, cells were transduced following activation as detailed previously (36, 39). Briefly, 2.5 × 10^5^ T cells were plated in 2 mL of complete RPMI per well in a 24-well plate containing transiently produced, replication-incompetent, RD114-pseudotyped retroviral particles immobilized on RetroNectin (Clontech Laboratories) and incubated for 48 hours at 37°C in 5% CO_2_.

### Vector copy-number enumeration

Primer/probe-FAM was designed to the Moloney murine leukemia virus–derived psi present in the pSFG backbone and purchased from Thermo Fisher Scientific. RNase P primer/probe-VIC/TAMRA mix (Applied Biosystems) was used for comparison. The vector copy number (VCN) measurement was performed as previously described (43). VCN calculation was performed using the 2^−ΔCt^ method (44).

### Flow cytometry

Transduction efficiency, synthetic receptor or GFP expression, T-cell immunophenotype, and detection of circulating cells in mouse peripheral blood were assessed by flow cytometry. A detailed list of all antibodies and fluorophore-conjugated ligands used can be found in Supplementary Table S2. Receptor surface expression was detected and measured using either incubation with His-tagged recombinant CD123 protein (Sino Biological) and secondary staining with anti–His-PE or anti–His-APC (BioLegend) or incubation with fluorophore-conjugated SNAP (New England Biolabs) or Halo (Promega) tags. For animal studies, peripheral blood was collected from the submandibular vein in living animals or extracted from the heart on necropsy. When indicated, the spleen and right hind leg (femur and tibia) were harvested from each mouse for the collection of splenocytes and bone marrow, respectively. Erythrocytes were lysed in single-cell suspensions with 1× RBC lysis buffer (Thermo Fisher Scientific). For *in vitro* studies and analysis of murine samples, cells were freshly stained in FACS buffer (PBS with 1% FBS) for 15 minutes at room temperature. Data were acquired using either a BD FACSCelesta or BD FACSymphony A5 flow cytometer and analyzed using FlowJo software (versions 10.8 and 10.9, RRID: SCR_008520).

### Phosphoflow

Intracellular staining and flow cytometric analysis were used to measure phosphoSTAT5 in T cells. Cells were cultured in cytokine-free media for at least 24 hours before the assay was performed. If indicated, cells were then plated on 200 ng/well of plate-bound rhCD123 and stimulated for 30 minutes at 37°C. Cells were fixed with Phosflow Fix Buffer I (BD Biosciences), followed by permeabilization with Phosflow Perm Buffer III (BD Biosciences) according to the manufacturer’s protocol. Cells were stained with STAT5(pY694)-Alexa Fluor 647 (Thermo Fisher Scientific). Data were collected on a BD FACSCelesta flow cytometer and analyzed using FlowJo software (versions 10.8 and 10.9, RRID: SCR_008520).

### ELISA

For cytokine detection (IL2, IL7, and IFNγ), 1 × 10^6^ T cells were plated with or without leukemia cell lines at a 1:1 effector-to-target (E:T) ratio in 2 mL of complete RPMI without exogenous cytokine supplementation for 24 hours. For the detection of *in vivo* cytokine secretion, mouse peripheral blood was obtained from the submandibular vein, and the plasma was collected after centrifugation of whole blood. ELISA was performed using the DuoSet ELISA kits (R&D Systems) according to the manufacturer’s instructions. Sample absorbance at 450 nm was read on a BMG CLARIOstar microplate reader.

### Western blot analysis

T cells were plated overnight in complete RPMI and starved of supplemental cytokine. Cells were next lysed in RIPA lysis buffer with protease (cOmplete) and phosphatase (PhosSTOP) inhibitor cocktails (MilliporeSigma) on ice. Total protein was quantified using a BCA protein quantification kit (Pierce). Electrophoresis was conducted using Novex WedgeWell 10% Bis-Tris Mini Gels (Thermo Fisher Scientific), and the protein was transferred to polyvinylidene difluoride membranes. Western blot analysis was performed with the following antibodies: rabbit anti–human pSTAT5 (Cell Signaling Technology), rabbit anti–human STAT5 (Cell Signaling Technology), and mouse anti–human GAPDH (Invitrogen).

### Time-resolved fluorescence energy transfer assay

For the detection of STAT5 phosphorylation using time-resolved fluorescence energy transfer (TR-FRET), STAT5 (Total) and STAT5 (Phospho-Tyr 694/699) TR-FRET assay kits (Cayman Chemical) were used. T cells were starved from cytokine for at least 24 hours before stimulation (if noted, with 10 ng/mL rhIL7 or on immobilized rhCD123 for 30 minutes). After stimulation, 2 × 10^5^ cells were plated in duplicate on a white, low-volume 384-well plate, lysed, and treated according to the manufacturer’s instructions. The assay was read using a BMG CLARIOstar microplate reader at 610 and 650 nm, and data were calculated as acceptor-to-donor emission signal (650 nm/610 nm × 1,000) as per the manufacturer’s direction.

### Incucyte proliferation assay

T cells expressing NLS.eGFP were plated in complete RPMI at 1 × 10^4^ cells per well in a 96-well, poly-D-lysine–coated, flat clear bottom plate in triplicate. The plate was incubated for 1 week in a humidified incubator at 37°C and 5% CO_2_ equipped with an Incucyte S3 live-cell imaging system. Phase-contrast and green fluorescence images were collected every 4 hours at four sites per well. Data were aggregated using Incucyte 2021A software, and then raw values were exported and analyzed using GraphPad Prism 10 (RRID: SCR_002798).

### Incucyte serial stimulation cytotoxicity assay

Acute myeloid leukemia (AML) target cell lines (MV-4-11 and Molm-13) engineered to express NLS.eGFP were used for long-term Incucyte cytotoxicity assays. Target cells were plated in coculture with T cells at a 1:1 E:T ratio (2 × 10^5^ total cells per condition) in 200 μL of complete RPMI on a flat-bottom, poly-D-lysine–coated 96-well plate in triplicate. The plates were scanned, and phase-contrast and green fluorescence images were detected every 4 hours, with acquisition of four images per well. Cocultures were stimulated with the addition of 1 × 10^5^ new target cells per well every 48 hours. Incucyte 2021A software was used to compile data and export raw data. Data were analyzed using GraphPad Prism 10 (RRID: SCR_002798).

### Metabolic assay

CAR-T cells were plated on rhCD123 (200 ng/well) at 1 × 10^6^ cells per well in a 24-well plate and 2 mL of complete RPMI for 24 hours. Extracellular flux assays were performed in a 96-well XF Extracellular Flux Analyzer (Agilent). For the mitochondrial stress test, cells (3 × 10^5^ per well, five replicates per condition) were plated in complete XF medium (nonbuffered RPMI 1640 containing 25 mmol/L glucose, 2 mmol/L L-glutamine, and 1 mmol/L sodium pyruvate) onto a poly-D-lysine–coated 96-well XF cell culture microplate. Cells were equilibrated for 45 minutes at 37°C in the absence of CO_2_. The oxygen consumption rate (OCR, in pmol/minutes) was measured under basal conditions and in response to 1 μmol/L oligomycin, 1.5 μmol/L FCCP, and 100 nmol/L rotenone + 1 μmol/L antimycin A (Sigma-Aldrich). Basal mitochondrial respiration was calculated by subtracting the OCR after treatment with rotenone and antimycin A from the baseline OCR measurements. Maximal mitochondrial respiration was calculated by subtracting the OCR after treatment with rotenone and antimycin A from the OCR measured following treatment with FCCP. Spare respiratory capacity was calculated by subtracting the basal mitochondrial respiration from the maximal mitochondrial respiration. For the glycolysis stress test, cells (3 × 10^5^ per well, five replicates per condition) were plated in XF medium without glucose onto poly-D-lysine–coated 96-well XF cell culture microplates. Cells were equilibrated for 45 minutes at 37°C in the absence of CO_2_. The extracellular acidification rate (ECAR) was measured under basal conditions and in response to 10 mmol/L glucose, 1 μmol/L oligomycin, and 50 mmol/L 2-deoxyglucose (2-DG; Sigma-Aldrich). The basal ECAR (corresponding to the nonglycolytic acidification rate) was determined, and glycolysis was calculated by subtracting the ECAR measured after the 2-DG injection from the ECAR measured following the injection of glucose. Glycolytic capacity was calculated by subtracting the ECAR measured after the 2-DG injection from the ECAR measured following the injection of oligomycin.

### Short-term cytotoxicity

ffLuc-expressing AML (MV-4-11 and Molm-13) or chronic myeloid leukemia (K-562, K562.CD123) cell lines were plated in coculture with T cells at indicated E:T ratios. After 18 hours of incubation, D-luciferin (Thermo Fisher Scientific) was added at a final concentration of 150 mg/mL and radiance measured using a BMG CLARIOstar microplate reader.

### Xenograft models

All animal studies were carried out under protocols approved by the Johns Hopkins Institutional Animal Care and Use Committee. Six- to eight-week-old NOD.*Cg-Prkdc*^*scid*^*Il2rg*^*tm1Wjl*^/SzJ (NOD/SCIDγ, NSG) mice were obtained from an internal colony that originated from The Jackson Laboratory. For leukemia studies, mice were injected via tail vein with 1 × 10^6^ ffLuc-expressing MV-4-11 cells in a total volume of 200 mL PBS. Six days later, mice were imaged using an IVIS Spectrum *in vivo* imaging system (PerkinElmer) following intraperitoneal injection of 100 mL of 150 mg/mL D-luciferin (Thermo Fisher Scientific). Data were analyzed using Living Image software (v. 4.7.3, 64-bit, PerkinElmer). Mice were assigned to treatment groups to normalize any differences in observed leukemia burden and were injected intravenously with T cells on the following day (day 7). For experiments without leukemia infusion, mice were injected with T cells on day 0 via tail vein. If indicated, mice received daily intraperitoneal injections of 5 μg of rhIL7 in 100 μL of PBS. Mice were monitored for a maximum of 100 days with weekly imaging, weight measurements, and clinical observation. Animals were sacrificed when humane endpoints were reached or at experiment end per protocol guideline.

### Proteomic analyses

Protein samples (90 mg each) were reduced with 50 mmol/L dithiothreitol in 10 mmol/L triethylammonium bicarbonate (TEAB) at 60°C for 45 minutes followed by alkylation with 100 mmol/L iodoacetamide in 10 mmol/L TEAB at room temperature in the dark for 15 minutes. A single-pot, solid-phase sample preparation protocol (SP3; ref. 45) using a mixture of Sera-Mag SpeedBeads (GE Healthcare) was used to remove detergents and other nonprotein contaminants. The proteins bound to beads were resuspended in 100 mL of 100 mmol/L TEAB and digested with 8 mg of trypsin/Lys-C (Pierce) overnight at 37°C. The digested peptides were separated from the magnetic beads using a magnetic tube holder and placed into a clean 0.5-mL Eppendorf centrifuge tube.

#### Isobaric mass tag labeling

Peptides in each of the 15 digested samples were labeled with a unique tandem mass tag (TMT) pro 16plex reagent (Thermo Fisher Scientific) according to the manufacturer’s instructions. All 15 TMT-labeled peptide samples were combined and dried by vacuum centrifugation.

#### Peptide fractionation

The combined TMT-labeled peptides (1,350 mg) were reconstituted in 100 μL of 10 mmol/L TEAB buffer and filtered through a Pierce Detergent Removal Column (Thermo Fisher Scientific) to remove excess TMT label, small molecules, and lipids. Peptides in the flow-through were diluted to 2 mL in 10 mmol/L TEAB in water and loaded on an XBridge C18 Guard Column (5 μm, 2.1 × 10 mm, Waters Corporation) at 250 μL/minutes for 8 minutes prior to fractionation on an XBridge C18 Column (5 μm, 2.1 × 100 mm column, Waters Corporation) using a 0% to 90% acetonitrile (ACN) in 10 mmol/L TEAB gradient over 85 minutes at 250 μL/minutes on an Agilent 1200 series capillary HPLC system with a microfraction collector. Eighty-four 250-μL fractions were collected and concatenated into 12 fractions (46).

#### Phosphopeptide enrichment

From each of the 12 fractions, 10% was extracted and pooled in sets of 3, yielding 4 unenriched fractions for total protein normalization. The remaining 90% was subjected to titanium dioxide (TiO_2_) phosphopeptide enrichment. Briefly, the remaining fractions were dried and brought up in a solution of 80% ACN, 5.0% trifluoroacetic acid (TFA), 1.25 mg 2,5-dihydroxybenzoic acid, and 0.5 mg TiO_2_ in 100 μL volume. The fractions were vortexed for 2 hours and then loaded onto stage tips constructed from Empore SDB-XC solid-phase extraction disks (3 M). The TiO_2_ concentrated on the head of the stage tip was then washed with 2 × 20 μL volumes of 80% ACN containing 5% TFA and 1 M glycolic acid, followed by a final rinse of 80% ACN and 0.1% TFA. The phosphopeptides were then eluted from TiO_2_ in a solution of 30% ACN and 3% NH_4_OH with two 20 μL aliquots, followed by a final elution with 60% ACN. The fractions were then neutralized with 20 μL of 10% TFA and dried.

#### Mass spectrometry

Peptides from the 4 unenriched fractions plus the 12 enriched fractions were brought up in 2% ACN and 0.1% formic acid and analyzed on Nano-LC-Orbitrap Fusion Lumos in OTOT mode (Thermo Fisher Scientific) interfaced with an EASY-nLC 1200 system. Peptides were separated using reversed-phase chromatography on an in-house packed 75 μm × 200 mm ReproSil-Pur-120-C18-AQ column 3 μm, 120 Å (Dr. Maisch) using 0.1% formic acid in water as mobile phase A and 0.1% formic acid in ACN as mobile phase B. The solvent gradient was as follows [time (minutes), %B]: (0, 0), (0.1, 7), (45.1, 20), (77.1, 32), (79.1, 100), (80.1, 100), (82.1, 0), and (90, 0). The mobile phase flow rate was held at 300 nL/minutes throughout the analysis. Eluting peptides were sprayed into the mass spectrometer through a 10-μm emitter tip (New Objective) at 2.6 kV. Survey scans of precursor ions were acquired from 400 to 1,600 m/z at 120,000 resolution at 200 m/z. MS/MS spectra were collected using data-dependent acquisition wherein precursor ions with a minimum intensity of 5e4 were isolated using the quadruple with an isolation of 0.7 m/z and subsequently fragmented using high-energy collisional dissociation (HCD) and an activation energy of 36. MS/MS spectra were collected at a resolution of 500,000 in the top-speed mode and a 3-second cycle time, and dynamic exclusion was activated for 15 seconds. Technical variation in ratios from our mass spectrometry analysis is less than 10% (47).

#### Proteomics data analysis

Raw mass spectrometry data were analyzed using MaxQuant (v2.4.2.0, RRID: SCR_014485; ref. 48). MS/MS spectra were searched against the UniProt human reference proteome (downloaded April 2023), which was supplemented with the CCR sequence. Carbamidomethylation of cysteine was set as a fixed modification, and variable oxidation of methionine, deamidation of asparagine and glutamine, and acetylation of the protein N-terminal were allowed. In the case of phosphopeptide-enriched samples, phosphorylation of serine, threonine, and tyrosine was set as an additional variable modification. Trypsin was specified as the digestion enzyme, and a maximum of two missed cleavages were allowed. TMT reporter ion intensities were corrected for isotopic impurities using the correction factors provided with the TMTpro kit. An FDR of 1% was used at both the peptide and protein levels. Decoy and contaminant proteins and peptides were removed from search results, and corrected reporter ion intensities were median-normalized. All further statistical analyses were conducted using Perseus (v1.6.12.0; ref. 49).

### Statistical analysis

Statistical analysis apart from composite group comparison was performed using GraphPad Prism 10 software (RRID: SCR_002798). Comparison of two groups was performed using unpaired *t* tests. Comparisons between greater than two groups were made with one- or two-way ANOVA and if comparison to a control group, Dunnett, or otherwise, Tukey correction. Survival was estimated using the Kaplan–Meier method, and differences in survival between groups were calculated by log-rank with Gehan–Breslow–Wilcoxon tests. The long-term cytotoxicity comparative killing rate was measured by fitting one-phase decay with least squares regression and constraint of time 0 at 1 to the equation *Y* = (Y0 – Plateau) × exp(−K × X) + Plateau. The 95% confidence intervals were calculated and plotted. To determine whether IL7R activation resulted in preferential expansion or retention of early memory T-cell subsets, we analyzed the compositional change in T-cell subsets between the four groups using the compositional data analytic method (50). Compositions of T-cell subsets (percentage of each subset) were transformed using an isometric log-ratio transformation, and then multivariate linear regression was performed with the treatment group as the independent categorical variable. Analyses were conducted in R (version 4.3.1) using the package “compositions” (51).

### Data availability

The raw and analyzed data generated during the study are available from the corresponding author for research purposes upon reasonable request. Proteomics data were uploaded to ProteomeXchange, project ID: PXD05483.

## Results

### T-cell activation downstream of an engineered CCR is similar to canonical IL7R activation

To compare T cells expressing our (CCR) to cells stimulated with extrinsic IL7, we designed vectors encoding our CCR or full-length IL7 (Fig. 1A). The CCR was encoded in a pSFG retroviral plasmid containing a 5′ long terminal repeat promoter and an IgHV leader sequence. The CCR itself was composed of an scFv binding the AML-associated antigen target CD123 (26292; ref. 38), an IgG4 hinge region, and the transmembrane and intracellular domains of IL7Rα (36). Each vector also included the coding sequence of eGFP with an N-terminal nuclear localization sequence to facilitate cell detection in imaging experiments and for measurement of vector transduction (Fig. 1A). Primary human T cells were stably transduced with these vectors. During initial activation and expansion, all cells were maintained with IL2 supplementation. CCR-T cells were compared with cells engineered with constitutive sIL7 and to cells grown with supplemental exogenous human recombinant IL7 (+IL7). Although the CCR contained an extracellular scFv to facilitate amplified signal upon target binding, we have previously found expression of this receptor to stimulate constitutive, tonic downstream signaling (36). We thus used this CCR to compare intrinsic with extrinsic IL7R activation via IL7 binding to the endogenous IL7R or CCR signaling. Using our vectors, all cell cohorts were efficiently modified [transduction efficiency measured on %GFP-positive cells: median (range), CCR: 66.7 (48.2–72.1)%; sIL7: 73.6 (55.1–79.2)%; GFP alone: 89.5 (82.6–94)%; Fig. 1B]. IL7 production in T cells engineered to secrete the cytokine was verified in tissue culture media and was quantified with a mean of 330.8 pg secreted from 1 × 10^6^ cells in 24 hours (range: 54.6–501.4 pg; Fig. 1C). The VCN per cell was measured with median values of 1.7 (range: 1.0–2.2) and 2.5 (range: 2.0–2.7) for CCR and sIL7 T-cell conditions, respectively (Supplementary Fig. S1A).

Having established successful CCR expression and constitutive sIL7 in T cells, we next tested the functionality of downstream IL7R signaling by measuring T-cell expansion, immunophenotype, STAT5 phosphorylation, phosphoprotein profile, and native IL7Rα (CD127) expression. Both CCR- and sIL7-expressing cells were able to expand *in vitro* without exogenous cytokine supplementation, whereas unmodified T cells did not, whether grown in IL2 alone or in the combination of IL2 and IL7 prior to cytokine withdrawal (Supplementary Fig. S1B). To determine whether tonic IL7R activation resulted in preferential expansion or retention of early memory T-cell subsets, we defined the immunophenotype of our cells during *ex vivo* culture. We found no significant differences between the four groups: CCR-expressing T cells (CCR), T cells constitutively secreting IL7 (sIL7), T cells treated with IL7 (+IL7), or control, untreated T cells (GFP-only; Fig. 1D; Supplementary Fig. S1C and S1D). IL7R activation was determined by measurement of STAT5 phosphorylation after cytokine starvation. All T-cell groups (CCR, sIL7, and IL7 supplementation) had increased STAT5 phosphorylation above that of untreated T cells reflective of ongoing CCR or IL7R downstream activation at the time of measurement (Fig. 1E). To determine whether the signaling pathway was saturated, we further treated cells from each group with 10 ng/mL IL7 for 30 minutes. All cells, with the exception of those secreting IL7, responded to IL7 treatment with increased STAT5 phosphorylation (Fig 1E). We tested CD127 surface receptor expression by flow cytometry and as expected, observed loss of native IL7Rα (CD127) expression in T cells with chronic IL7 exposure [%CD127^+^: median (range), CCR: 62.0 (41.2–68.6)%; sIL7: 13.3 (7.3–23.2)%; +IL7: 39 (34.3–46.1)%; no treatment: 60.4 (44.6–73.60)%; Fig. 1F and G]. To further define the signaling pathway activated downstream of the CCR compared with that resulting from canonical IL7/IL7R binding, we performed whole-phosphoproteomic analysis on T cells expressing the CCR and on unmodified cells treated with or without IL7 for 30 minutes prior to lysis. The scFv of the CCR was strongly detected at the whole-protein level in transduced cells (Supplementary Fig. S1E), with relevant IL7R pathway proteins also detected (Fig. 1H). Phosphorylation of STAT5A and STAT5B was observed in CCR and IL7 treatment conditions, although there is sequence identity in the phosphopeptides defining STAT5B pY699 and STAT5A pY694 (Fig. 1H). Principal component analysis of each sample was notable for strong clustering by T-cell donor, which unfortunately confounded our planned unbiased whole-phosphoproteomic analysis (Supplementary Fig. S1F and S1G). In summary, engineering of primary human T cells for intrinsic IL7R signaling pathway activation via CCR expression or constitutive sIL7 is possible, and these modifications effectively stimulate the similar downstream signaling. Notably, we verified that T cells chronically secreting IL7 and thereby chronically exposed to it downregulate CD127, making them unresponsive to extrinsic IL7 treatment.

### IL7 pathway activation supports T-cell survival and proliferation *in vivo*

**Figure 2:**
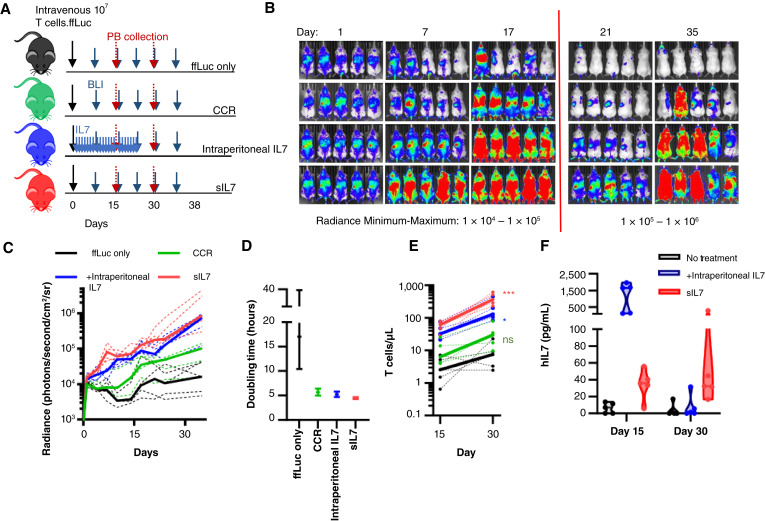
T cells with IL7 pathway activation expand and circulate *in vivo*. **A,** Schematic of experimental design. NSG mice were injected on day 0 with 10^7^ T cells expressing a ffLuc reporter. BLI was conducted biweekly, and peripheral blood collected on days 15 and 30. One group of mice was treated with ffLuc-only–modified T cells and intraperitoneal injections of IL7 daily from day 1 to 21. **B,** BLI monitoring T-cell expansion. **C,** Quantified radiance per mouse. Thick solid line, median BLI; dotted lines, individual values per mouse. **D,** Calculated doubling time of T-cell products with 95% confidence interval represented. **E,** T cells (CD3^+^GFP^+^) detected in peripheral blood on days 15 and 30 after injection. Thick solid line, median; dotted lines, individual values per mouse. Comparison between treatment cohorts on day 30 vs. no treatment: *, *P* < 0.05; ***, *P* < 0.001. **F,** Concentration of human IL7 in murine plasma detected with ELISA. For the intraperitoneally injected mice on day 15, blood was collected 1 hour after IL7 injection. (*n* = 5 mice per group). hIL7, human IL7; NSG, NOD/SCIDγ; NT, nontransduced; PB, peripheral blood.

To test whether the observed autonomous survival (Supplementary Fig. S1B) of our engineered cells would translate to T-cell expansion and survival following adoptive transfer, we injected CCR-expressing, IL7-secreting, or ffLuc-modified only human T cells into immunodeficient mice (Fig. 2A). We included a cohort of mice that received daily intraperitoneal injections of human IL7 for 21 days to mimic treatment of patients with systemic supportive IL7. All T cells were engineered to have constitutive ffLuc expression to allow for noninvasive monitoring of T-cell proliferation. T cells expressing IL7 (sIL7) or the CCR were transduced sequentially with ffLuc-containing retroviral vectors and then with the indicated IL7 or IL7R constructs. We found that all cohorts with IL7 pathway activation had continuous expansion of transferred cells for the duration of our experiment, with observed T-cell proliferative rates significantly faster than that of the control cohort (Fig. 2B–D). Monitoring of peripheral blood revealed differences in detectable numbers of circulating T cells at day 30 after injection (Fig. 2E). Relative T-cell expansion patterns were reflected in the numbers of T cells in the bone marrow and spleen of animals at necropsy (day 38, Supplementary Fig. S2A and S2B). Circulating IL7 was detectable in the peripheral blood of mice injected with T cells secreting IL7 and in those actively treated (days 1–21) with intraperitoneal IL7 (Fig. 2F). CD127 expression was measured on peripherally circulating T cells on days 15 and 30. CCR-expressing T cells retained CD127 expression, whereas IL7-secreting or exogenously treated cells lost expression (Supplementary Fig. S2C and S2D), consistent with our prior *in vitro* data (Fig. 1F and G). Interestingly, with clearance of intraperitoneally injected IL7 (day 30), CD127 expression returned, highlighting the dynamic nature of CD127 regulation (Supplementary Fig. S2C and S2D).

### CCR and CAR coexpression maintains individual receptor functionality

**Figure 3:**
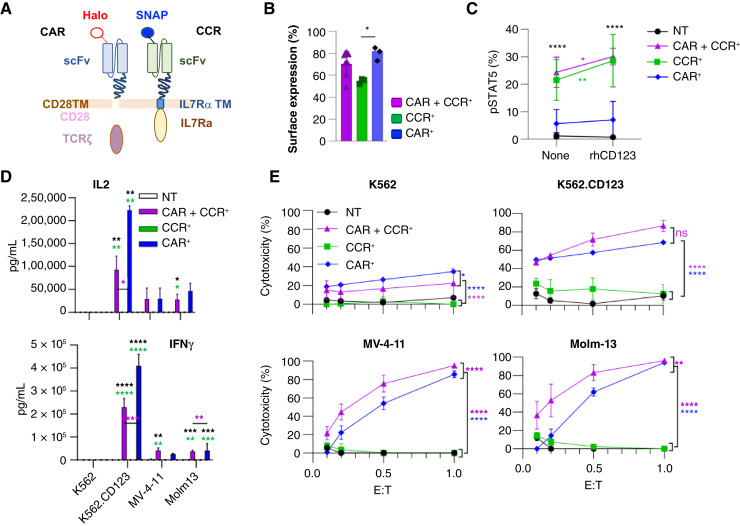
CD123-targeted CAR and CCR can be coexpressed with maintained functionality. **A,** Schematic showing CAR and CCR structures. **B,** Transduction efficiency measured using flow cytometric staining of Halo and SNAP tags. *n* = 3 to 6 unique T-cell donors, unless noted no significant difference between groups. **C,** Measurement of phosphorylated STAT5 percentage in cytokine starved, engineered T cells measured with and without activation on the immobilized rhCD123 target. *n* = 3 to 4 unique T-cell donors, comparison of CCR + CAR^+^ and CAR^+^ to NT (black) and with stimulation (colored). **D,** Soluble IL2 and IFNγ measured in the supernatant following coculture of unmodified (NT), CCR^+^, CAR^+^, or CCR + CAR^+^ T cells with CD123-negative (K562) and CD123-positive (K562.CD123, MV-4-11, and Molm-13) targets. *n* = 3 to 6 unique T-cell donors; data represented as mean ± SD. Significance noted is in comparison to NT (black asterisks) and/or CCR^+^ cells (green asterisks) or as noted. NT vs. CCR^+^ comparison was nonsignificant in all instances. **E,** Bioluminescence-based cytotoxicity assays performed using K562, K562.CD123, MV-4-11, and Molm-13 stably expressing ffLuc; *n* = 3 to 5 donors. For **B–E,** *, *P* < 0.05; **, *P* < 0.01; ***, *P* < 0.001; ****, *P* < 0.0001. Unless noted, comparisons were nonsignificant. NT, nontransduced.

Although we have previously shown enhanced T-cell proliferation and sustained antitumor activity in engineered, antigen-specific T cells expressing our CD123-targeted CCR (36), we had not tested whether our CCR would interfere with the function of a cytotoxic CAR and vice versa. We therefore coexpressed the CCR with a second-generation anti-CD123 CAR that contained an alternate scFv that binds a unique CD123 epitope (32716; ref. 38), CD28 costimulatory, and CD3ζ-activating domains (Fig. 3A). We added N-terminal SNAP (52, 53) and Halo (54) tags to each receptor to facilitate unique detection on the cell surface and verified that the addition of these tags did not interfere with either surface expression or target binding (Supplementary Fig. S3). The surface expression of the artificial receptors in CCR + CAR^+^ cells [median (range): 72 (49–81)%] did not significantly differ from that of cells expressing the CCR alone [56 (52–56)%], although we found increased expression of the CAR alone [82 (73–85)%] versus the CCR (Fig. 3B). We also found that after cytokine starvation, CCR^+^ and CCR + CAR^+^ T cells had equal amounts of measurable STAT5 activation [%pSTAT5^+^: median (range), CCR + CAR^+^: 26.4 (21.5–30.9)%; CCR^+^: 22 (12.1–30)%]. Both engineered T-cell types exhibited a higher percentage of cells positive for pSTAT5 than that of CAR^+^ cells [4.2 (1.4–11.3)%, *P* < 0.0001] or unmodified T cells [0.9 (0.16–2.7)%, *P* < 0.0001; Fig. 3C]. STAT5 activation did not differ between unmodified or CAR^+^-T cells. Stimulation of cells with immobilized rhCD123 significantly increased these levels in cells expressing the CCR only [CCR + CAR^+^: 32.0 (25.7–32.4)%, *P* < 0.05; CCR^+^: 26.8 (19.3–41.4)%, *P* < 0.01; CAR^+^: 4.2 (2.2–14.7)%; NT: 0.65 (0.2–1.4)%, Fig 3C]. As expected, antigen-specific cytokine secretion and cytotoxic activity were evident only in cells expressing the CAR (Fig 3D and E). CAR^+^ cells also expressing the CCR demonstrated similar antigen-specific activation with increased cytotoxicity against CD123^+^ AML cell lines (Fig. 3D and E).

**Figure 4:**
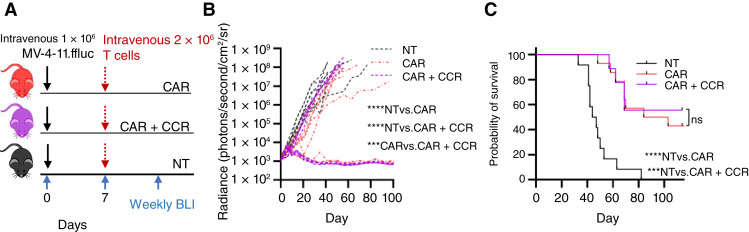
CCR expression does not diminish CAR T-cell antitumor activity. **A,** Schematic of the xenograft model. On day 0, NSG mice were injected via tail vein with 1 × 10^6^ CD123^+^ MV-4-11.ffLuc cells. In treatment groups, 2 × 10^6^ T cells were administered on day 7. Cohorts: unmodified (NT), CD28.ζ CAR, and CD28.ζ CAR + IL7Rα CCR-T cells. **B,** Leukemia proliferation was monitored with BLI, and whole-animal radiance was recorded (photons/second/cm^2^/sr). Comparison of radiance in each group was performed following log transformation of each measured value. **C,** Kaplan–Meier survival analysis of MV-4-11 xenografts (*n* = 9 mice per condition; two independent experiments; median survival: NT: 45.5 days; CAR: 93.5 days; CAR + CCR: undefined; ***, *P* < 0.001; ****, *P* < 0.0001). NSG, NOD/SCIDγ; NT, nontransduced.

We next used a xenograft model of human AML to test the potential benefit of CCR and CD28.ζ CAR coexpression (Fig. 4A). NOD/SCIDγ mice were injected with MV-4-11.ffLuc, a CD123^+^ AML cell line engineered with constitutive ffLuc expression. Treatment groups received T cells either expressing the CD123-targeted cytotoxic CAR alone or T cells coexpressing the CAR and CCR. Control mice received unmodified T cells. Of mice treated with T cells harboring CAR + CCR coexpression, 56% (5/9) had rapid tumor clearance that was sustained, in comparison to 33% (3/9) of mice treated with T cells expressing CAR only. Thus, CCR expression improved *in vivo* antitumor activity of CAR-T cells targeting CD123 in this model (Fig. 4B). Log transformation of the quantitative BLI to account for exponential leukemia proliferation and eliminate bias reveals that both engineered T-cell treatment groups have a significantly different overall leukemia proliferation from the unmodified T-cell control group (comparison of nontransduced vs. CAR: *P* < 0.0001, nontransduced vs. CAR + CCR: *P* < 0.0001). A comparison of engineered T-cell groups is also significantly different (CAR vs. CAR + CCR: *P* = 0.0003). All treated mice demonstrated improved survival over the control group (*P* < 0.001), although the median survival was not defined in the CCR + CAR group because more than half of the mice were cured of disease at the experimental endpoint (median survival: unmodified T cells: – 45.5 days; CAR: – 93.5 days; CCR + CAR: undefined; Fig. 4C).

### CCR hinge modification eliminates tonic signaling but does not improve antigen specificity

**Figure 5:**
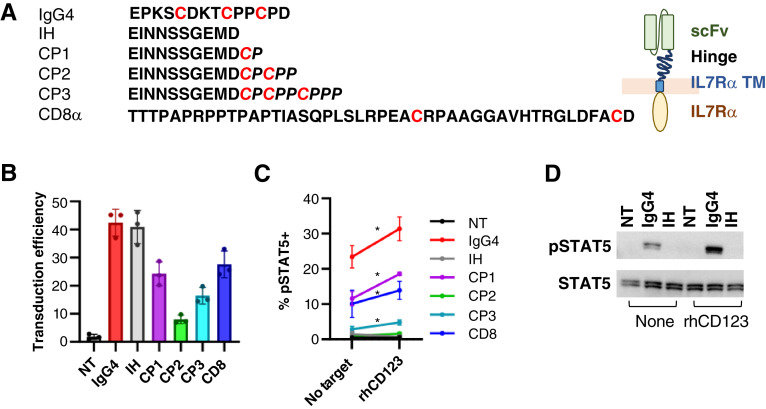
Hinge modifications do not improve antigen specificity of CD123-targeted chimeric IL7Rα. **A,** Amino acid sequences of hinge regions used in functional studies. **B,** Transduction efficiency measured using his-tagged rhCD123 binding of engineered receptors and flow cytometry. *n* = 3 unique T-cell donors. **C,** Quantification of phosphorylated STAT5 percentage in CCR-T cells measured with and without activation on the immobilized rhCD123 target. *n* = 3 unique T-cell donors, comparison between pSTAT5% in cytokine-starved cells with or without rhCD123 activation; *, *P* < 0.05. **D,** Representative Western blot testing IgG4 vs. IH STAT5 activation with and without immobilized rhCD123. NT, nontransduced.

Given our observed cytokine-independent, autonomous proliferation and survival in cells expressing either the CCR or with constitutive sIL7, we attempted to improve the antigen specificity of downstream IL7R signaling by introducing alternate hinge structures into the CCR (Fig 5A). Our existing hinge was derived from the human IgG4 molecule. Though not the optimal IL7R structure (heterodimerization of CD127 and γc), homodimerization of CD127 can occur and is stabilized by disulfide bridge formation between embedded cysteine residues (37). We therefore hypothesized that homodimerization may be contributing to tonic CCR activation and compared our IgG4 hinge-containing CCR to a CCR with a hinge containing no cysteines [inert hinge (IH)] and including a series of juxtamembrane cysteines (CP1, CP2, and CP3) conceived to correspond to each cysteine in the IgG4 hinge. We also tested a hinge derived from the human CD8α receptor as this hinge is commonly used in clinical CAR designs (55). Surface expression of CCRs containing novel hinges was variable [%CCR^+^ cells: median (range), IgG4: 45.1 (36.9–45.2)%; IH: 42.0 (36.9–45.2)%; CP1: 23.7 (20.2–28.7)%; CP2: 7.3 (6.9–9.7)%; CP3: 15.0 (14.4–19.8)%; CD8: 25.6 (24.1–33.0)%; Fig. 5B] and correlated with the percentage of cells with activated STAT5 phosphorylation detected by intracellular flow cytometry (Fig. 5C). Notably, incorporation of CD8α, CP1, and IgG4 hinges in the CCR resulted in STAT5 activation at baseline above that seen in unmodified T cells. Plating cells on rhCD123, the target of the extracellular binding domain of each CCR, enhanced STAT5 phosphorylation for the IgG4, CP1, CP2, and CD8 hinge-containing constructs (Fig. 5C). We verified flow results using Western blot analysis for the IH and IgG4 hinge-containing constructs, thereby confirming the constitutive, but antigen-responsive, nature of the IgG4-containing CCR (Fig. 5D).

### IL7Rα domain signaling is dependent on membrane proximity

**Figure 6:**
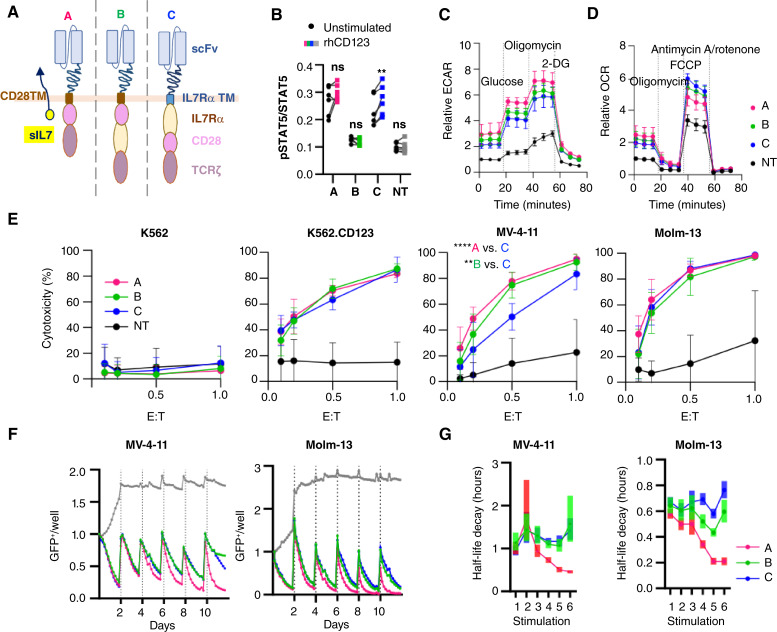
Single-chain IL7Rα signaling stimulates STAT5 activation in CAR-T cells. **A,** Schema of the compared T-cell conditions. A: CAR and sIL7; B: CAR with the distal IL7Rα domain; C: CAR with the proximal IL7Rα domain. **B,** Levels of STAT5 phosphorylation before and after exposure to plate-bound recombinant antigen (rhCD123) for 30 minutes, measured with a TR-FRET assay. Comparison between conditions with and without rhCD123 stimulation. *n* = 3 donors, performed in duplicate. **C,** Relative ECAR and (**D**) relative OCR of T cells measured after 24-hour rhCD123 stimulation in cytokine-free media. *n* = 4 individual donors tested in five replicates per assay. For each donor, normalization of all values to the baseline (NT control) was performed. **E,** Cytotoxic activity measured by luminescence-based assay. CD123-negative K562 cell line and NT T cells included as controls. *n* = 4 T-cell donors, E:T. Comparison of cytotoxicity of engineered T cells against all CD123-expressing targets with that from NT T cells was very significant (*P* < 0.0001 in K562.CD123, MV-4-11, and Molm-13). Differences between engineered T cells noted only with MV-4-11 target. ****, *P* < 0.0001; **, *P* < 0.01. **F,** Long-term cytotoxicity measured with six 48-hour serial stimulations with indicated NLS eGFP-modified AML cell lines with initial plating at 1:1 E:T (*n* = 4 individual donors in technical triplicate each normalized to timepoint 0). **G,** Half-life decay of each target cell line calculated at each stimulation. Shaded bars, 95% confidence intervals for decay calculated at each timepoint. NT, nontransduced.

Because of our inability to eliminate tonic signaling while retaining functionality, we tested the addition of an IL7Rα domain within our existing, reliably antigen-specific, cytotoxic CAR structure (Fig. 6A). We compared the functionality of T cells engineered with CCR–CAR hybrid receptors with that of T cells engineered for dual constitutive sIL7 and CD28.ζ CAR expression (Fig. 6A). Expression of these constructs was high in all cases [median (range), CAR + sIL7: 93.7 (82.5–96.3); CAR + distal IL7Rα domain, CD28.IL7R.ζ: 83.8 (69–93); CAR + proximal IL7Rα domain, IL7R.CD28.ζ: 70.4 (65–79.1); Supplementary Fig. S4A]. sIL7 was confirmed in CAR-T cells modified to express the cytokine with a mean of 189 pg secreted from 1 × 10^6^ cells in 24 hours (range: 72.6–316.2; Supplementary Fig. S4B). TR-FRET was used to measure baseline and antigen-stimulated STAT5 phosphorylation in engineered cells. IL7R signal transduction was evident in CAR-T cells secreting IL7 and in cells with the IL7Rα domain proximal to the membrane within their expressed CAR (Fig. 6B). These two T-cell types had increased levels of basal activation compared with unmodified T cells. Phosphorylated STAT5 was not elevated when the IL7Rα domain was distal to the CD28 domain. Responsiveness of the IL7Rα domain to antigen stimulation was only observed in membrane proximal IL7R.CD28.ζ T cells (Fig. 6B). The definition of T-cell subsets using surface receptor expression during *ex vivo* expansion did not reveal an altered subset distribution as compared with unmodified T cells in any condition (Supplementary Fig. S4C). We examined the metabolic state of the T cells after plate-bound antigen stimulation in cytokine-free media and found that all engineered conditions were more metabolically active than unmodified cells. Engineered T cells had a higher nonglycolytic basal ECAR and higher glycolysis after glucose addition than unmodified cells (Fig. 6C; Supplementary Fig. S4D). Mitochondrial metabolic activity as measured by the OCR was not different from that of unmodified cells at baseline, but the sIL7- and IL7R.CD28.ζ-expressing cells had increased maximal respiration (Fig. 6D; Supplementary Fig. S4E).

All CAR-expressing T cells retained antigen-specific cytotoxic activity across a wide range of E:T ratios in short-term coculture assays (Fig. 6E). Of note, short-term cytotoxic activity of T cells expressing the construct with membrane-proximal IL7Rα (IL7R.CD28.ζ) was reduced specifically against the target cell line MV-4-11 when compared with the other two constructs (Fig. 6E). Antitumor activity in long-term *in vitro* serial stimulation assays showed maintained cytotoxicity against two CD123^+^ AML target cell lines over six stimulations (Fig. 6F; Supplementary Fig. S5A and S5B). The IL7-secreting construct maintained quicker cell killing to experiment end as compared with CAR conditions with integrated IL7Rα domains (Fig. 6G). In summary, adding an IL7Rα domain to a cytotoxic CAR can add functionality, but we find in our model systems that this functionality is dependent on the physical location of the domain, with proximity to the cell membrane being critical to achieve the best IL7Rα downstream activity.

### IL7R signaling does not improve CAR T-cell activity versus AML *in vivo*

**Figure 7:**
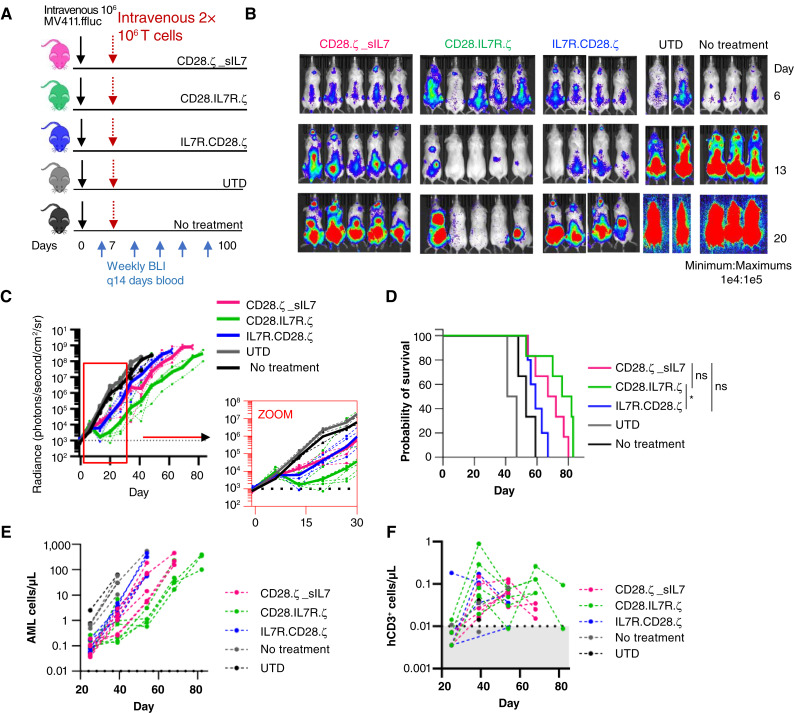
Single-chain proximal IL7Rα CAR-T cells have decreased *in vivo* antileukemia activity. **A,** Schematic of the xenograft model. On day 0, NSG mice were injected via tail vein with 1 × 10^6^ CD123^+^ MV-4-11.ffLuc cells. In treatment groups, 2 × 10^6^ CAR^+^ T cells were administered via tail vein on day 7. Cohorts: unmodified (NT, *n* = 2), CD28.ζ + sIL7 (*n* = 6), CD28.IL7R.ζ (*n* = 6), and IL7R.CD28.ζ (*n* = 5) T cells. Untreated mice (*n* = 3) served as controls. **B,** Representative mouse pictures of leukemia proliferation monitored with BLI. **C,** Radiance. Dotted lines, individual mice; solid line, median of each cohort. **D,** Kaplan–Meier survival analysis; comprehensive curve comparison; *P* < 0.0001; NT vs. CD28.ζ_sIL7, vs. CD28.IL7R.ζ, and vs. IL7R.CD28.ζ: all **, *P* < 0.01 individual comparisons. CD28.IL7R.ζ vs. IL7R.CD28.ζ: *, *P* < 0.01. **E,** AML and (**F**). T-cell counts detected in peripheral blood of mice and measured by flow cytometry. No significant differences found between groups. NSG, NOD/SCIDγ; NT, nontransduced.

We then evaluated the IL7-secreting and hybrid CCR–CAR T cells as AML treatments *in vivo*. We chose a noncurative treatment dose of T cells in order to determine whether the addition of IL7R downstream activation would promote persistent antitumor activity of the infused T cells (Fig. 7A). T cells were activated and engineered as in prior experiments and were maintained in IL2. IL7 was not used to supplement any T-cell condition in this experiment. Control T cells were not transduced. As expected, all T cells expressing AML-directed cytotoxic CARs displayed antitumor activity, even at the relatively low dose of 2 × 10^6^ cells infused per mouse (Fig. 7B–D). AML cells detected per mL of peripheral blood confirmed leukemic proliferation analogous to that observed using BLI (Fig. 7E). Measurement of CD3^+^ T cells in peripheral blood specimens did not reveal greater expansion or longer persistence of any T-cell treatment group (Fig. 7F). T cells expressing a CAR containing a distal IL7Rα domain (CD28.IL7R.ζ) had superior leukemia control *in vivo* when compared with the T cells expressing a CAR with a proximal IL7Rα domain (IL7R.CD28.ζ) and with CAR-T cells secreting IL7 (Fig. 7B–D). All engineered T-cell treatment groups had improved overall survival compared with mice treated with unmodified cells or with those that received no treatment (median survival: no treatment: 53 days; untransduced (UTD): 48 days; CD28.ζ CAR with secreted IL7: 69.5 days; CD28.IL7R.ζ: 79 days; IL7R.CD28.ζ: 59 days, *P* < 0.0001; Fig. 7D). Therefore, despite IL7R pathway activation in CAR-T cells secreting IL7 and in CAR-T cells with a membrane proximal IL7Rα domain, cells expressing a CD28-membrane proximal CAR had more powerful antitumor activity that led to the best survival in a mouse model of human AML.

## Discussion

Our approach equipped CAR-T cells with a constitutive, cell-intrinsic, signal 3. These data show that engineering a CCR with an extracellular AML-targeted scFv adds functionality downstream of the IL7R signaling domain to T cells, whereas coexpression with or within a CAR maintains target-specific cytotoxicity. Interestingly, we discovered that when embedded in the CAR molecule, spatial localization of the cytokine receptor signaling domain plays an important role in its signal transduction intensity and quality, a phenomenon also observed by others in relation to cytotoxic CAR-activating domains (56). It is likely that the subcellular location of CAR signaling domains is optimal when closely approximating that of native receptor structures. When the IL7Rα domain was located proximal to the cell membrane, it more effectively stimulated STAT5 activation. It is possible that distal localization stimulated lower intensity signaling below our threshold of detection, although this positioning may be too far physically removed from necessary intracellular signaling partners to be effective. Similarly, proximal IL7Rα localization may interfere with CAR cytotoxic functionality, as evidenced in our *in vitro* and *in vivo* studies. Although examined in a different context, membrane proximity of the CD3ζ chain as a component of the CAR structure has been found to strongly influence functional activity (56). On the other hand, it may be that different transmembrane domains (as in our CAR structures) affect synthetic receptor stability, conformation, or transactivation via interaction with other membrane elements. Other groups have shown this, with variable activation downstream of the CAR dependent on transmembrane elements (57–59). Future efforts will attempt to dissect the relative import of each modular CAR and CCR element, with a goal of optimal activation and antigen specificity.

Our engineered CCR exhibits antigen-independent, but responsive, constitutive activity that we were unable to improve with rational structural engineering of the hinge domain. Phosphoproteomic analysis was illustrative of increased activation as measured by STAT5A and STAT5B phosphorylation. We had not only hoped to discover a unique activation pattern resultant from the stimulation of the CCR but also planned to detail the pattern of downstream phosphorylation distinctly related to association of the IL7Rα chain with the common γ-chain. Ultimately, significant differences in the three evaluated T-cell donor phenotypes precluded further detailed analysis. We are designing engineering and activation approaches to continue to study CCR downstream activation.

Although our T cells secreting IL7 were effective at maintaining high levels of measurable T-cell activation, sIL7 from these cells is nonspecific and therefore uncontrolled. We are wary that uncontrolled sIL7 may ultimately reach toxic levels *in vivo*, especially in the local microenvironment. We additionally found that cells chronically exposed to high levels of IL7 downregulated CD127, making them impervious to dynamic IL7 levels absent from their own production. Given these negative characteristics, we believe that the provision of T cells with specific intracellular activation to be superior to uncontrolled cytokine secretion. We continue to seek a method that maintains the combination of powerful, specific downstream IL7R activation and efficient anti-AML activity *in vivo*. To this end, we believe there to be multiple advantages to expression of a single-chain molecule with embedded IL7R signaling. With a single chimera, functional domains are colocalized, which may enhance the efficiency of interactions dependent on spatial proximity. This is essential in CAR-T cells, in which activation greatly depends on antigen binding, receptor colocalization, and clustering, all which require precise molecular interactions (60, 61). In addition, the use of a single-chain expression strategy decreases transgenic size, which is likely to increase vector packaging, and cellular expression. Overall, a single-chain chimeric receptor is functionally and practically superior to planned expression of two separate molecules.

When considering our summative data, we find the capability of tumor-specific T cells to rapidly clear disease to be the primary determinant of treatment outcome in our animal studies. Similarly, therapy for AML is basically designed to eliminate bulk disease. However, it is well appreciated that achieving a balance between rapid, powerful antitumor activity and long-term disease surveillance is of the utmost importance for patients, probably because of persistent quiescent leukemic stem cells protected from chemotherapy and immunologic attack in the bone marrow niche (62). The need for CAR T-cell exposure that is initially high but also prolonged to cure leukemia has been validated in several clinical trials and subsequent analyses (7, 8, 63). Although we saw no differences in the compositional makeup of T-cell subsets during *ex vivo* culture, we are evaluating the evolution of T-cell populations following antigen exposure, as we hypothesize that the responsiveness of IL7R-mediated signaling to antigen binding may support the retention of characteristically memory-like T cells. Xenograft models of AML do not fully recapitulate the human condition because of faulty leukemic stem cell, cytokine, and marrow stromal cell interactions. We believe that a T-cell metabolic state that supports anticancer activity in a hypoxic, nutrient-deplete, acidotic environment akin to the marrow space in which the leukemic stem cells reside to be necessary for full disease clearance. We are encouraged to have seen increased spare respiratory capacity and maximal mitochondrial respiration in cells activated by membrane proximal IL7Rα domain signaling. All CAR-expressing T cells also had increased glycolysis above that of unmodified T cells, which suggests that the IL7R pathway–activated T cells may have improved and effective antitumor activity in the metabolically unfavorable leukemia microenvironment. We are exploring the use of alternate model systems that may allow experimental testing of altered T-cell metabolic states.

## Supplementary Material

Figure S1 Supplementary Figure 1

Figure S2 Supplementary Figure 2

Figure S3 Supplementary Figure 3

Figure S4 Supplementary Figure 4

Figure S5 Supplementary Figure 5

Supplementary Tables Supplemental Table 1 and 2

## References

[1] Laetsch TW , Maude SL, Rives S, Hiramatsu H, Bittencourt H, Bader P, . Three-year update of tisagenlecleucel in pediatric and young adult patients with relapsed/refractory acute lymphoblastic leukemia in the ELIANA trial. J Clin Oncol 2023;41:1664–9. 36399695 10.1200/JCO.22.00642 PMC10022844

[2] Curran KJ , Margossian SP, Kernan NA, Silverman LB, Williams DA, Shukla N, . Toxicity and response after CD19-specific CAR T-cell therapy in pediatric/young adult relapsed/refractory B-ALL. Blood 2019;134:2361–8. 31650176 10.1182/blood.2019001641 PMC6933289

[3] Gardner RA , Finney O, Annesley C, Brakke H, Summers C, Leger K, . Intent-to-treat leukemia remission by CD19 CAR T cells of defined formulation and dose in children and young adults. Blood 2017;129:3322–31. 28408462 10.1182/blood-2017-02-769208 PMC5482103

[4] Lee DW , Kochenderfer JN, Stetler-Stevenson M, Cui YK, Delbrook C, Feldman SA, . T cells expressing CD19 chimeric antigen receptors for acute lymphoblastic leukaemia in children and young adults: a phase 1 dose-escalation trial. Lancet 2015;385:517–28. 25319501 10.1016/S0140-6736(14)61403-3 PMC7065359

[5] Turtle CJ , Hanafi L-A, Berger C, Gooley TA, Cherian S, Hudecek M, . CD19 CAR-T cells of defined CD4^+^:CD8^+^ composition in adult B cell ALL patients. J Clin Invest 2016;126:2123–38. 27111235 10.1172/JCI85309 PMC4887159

[6] Park JH , Rivière I, Gonen M, Wang X, Sénéchal B, Curran KJ, . Long-term follow-up of CD19 CAR therapy in acute lymphoblastic leukemia. N Engl J Med 2018;378:449–59. 29385376 10.1056/NEJMoa1709919 PMC6637939

[7] Maude SL , Laetsch TW, Buechner J, Rives S, Boyer M, Bittencourt H, . Tisagenlecleucel in children and young adults with B-cell lymphoblastic leukemia. N Engl J Med 2018;378:439–48. 29385370 10.1056/NEJMoa1709866 PMC5996391

[8] Finney OC , Brakke HM, Rawlings-Rhea S, Hicks R, Doolittle D, Lopez M, . CD19 CAR T cell product and disease attributes predict leukemia remission durability. J Clin Invest 2019;129:2123–32. 30860496 10.1172/JCI125423 PMC6486329

[9] Jameson SC , Masopust D. Understanding subset diversity in T cell memory. Immunity 2018;48:214–26. 29466754 10.1016/j.immuni.2018.02.010 PMC5863745

[10] Xu Y , Zhang M, Ramos CA, Durett A, Liu E, Dakhova O, . Closely related T-memory stem cells correlate with in vivo expansion of CAR.CD19-T cells and are preserved by IL-7 and IL-15. Blood 2014;123:3750–9. 24782509 10.1182/blood-2014-01-552174 PMC4055922

[11] Pearce EL , Poffenberger MC, Chang C-H, Jones RG. Fueling immunity: insights into metabolism and lymphocyte function. Science 2013;342:1242454. 24115444 10.1126/science.1242454 PMC4486656

[12] Sommermeyer D , Hudecek M, Kosasih PL, Gogishvili T, Maloney DG, Turtle CJ, . Chimeric antigen receptor-modified T cells derived from defined CD8^+^ and CD4^+^ subsets confer superior antitumor reactivity in vivo. Leukemia 2016;30:492–500. 26369987 10.1038/leu.2015.247 PMC4746098

[13] Biasco L , Scala S, Basso Ricci L, Dionisio F, Baricordi C, Calabria A, . In vivo tracking of T cells in humans unveils decade-long survival and activity of genetically modified T memory stem cells. Sci Transl Med 2015;7:273ra13. 10.1126/scitranslmed.3010314 25653219

[14] Arcangeli S , Bove C, Mezzanotte C, Camisa B, Falcone L, Manfredi F, . CAR T cell manufacturing from naive/stem memory T lymphocytes enhances antitumor responses while curtailing cytokine release syndrome. J Clin Invest 2022;132:e150807. 35503659 10.1172/JCI150807 PMC9197529

[15] Schluns KS , Kieper WC, Jameson SC, Lefrançois L. Interleukin-7 mediates the homeostasis of naïve and memory CD8 T cells in vivo. Nat Immunol 2000;1:426–32. 11062503 10.1038/80868

[16] Li J , Huston G, Swain SL. IL-7 promotes the transition of CD4 effectors to persistent memory cells. J Exp Med 2003;198:1807–15. 14676295 10.1084/jem.20030725 PMC2194161

[17] Chen D , Tang T-X, Deng H, Yang X-P, Tang Z-H. Interleukin-7 biology and its effects on immune cells: mediator of generation, differentiation, survival, and homeostasis. Front Immunol 2021;12:747324. 34925323 10.3389/fimmu.2021.747324 PMC8674869

[18] Bradley LM , Haynes L, Swain SL. IL-7: maintaining T-cell memory and achieving homeostasis. Trends Immunol 2005;26:172–6. 15745860 10.1016/j.it.2005.01.004

[19] Rochman Y , Spolski R, Leonard WJ. New insights into the regulation of T cells by gamma(c) family cytokines. Nat Rev Immunol 2009;9:480–90. 19543225 10.1038/nri2580 PMC2814538

[20] Mazzucchelli R , Durum SK. Interleukin-7 receptor expression: intelligent design. Nat Rev Immunol 2007;7:144–54. 17259970 10.1038/nri2023

[21] Kieper WC , Tan JT, Bondi-Boyd B, Gapin L, Sprent J, Ceredig R, . Overexpression of interleukin (IL)-7 leads to IL-15-independent generation of memory phenotype CD8^+^ T cells. J Exp Med 2002;195:1533–9. 12070281 10.1084/jem.20020067 PMC2193553

[22] Melchionda F , Fry TJ, Milliron MJ, McKirdy MA, Tagaya Y, Mackall CL. Adjuvant IL-7 or IL-15 overcomes immunodominance and improves survival of the CD8^+^ memory cell pool. J Clin Invest 2005;115:1177–87. 15841203 10.1172/JCI23134 PMC1074679

[23] Schluns KS , Lefrançois L. Cytokine control of memory T-cell development and survival. Nat Rev Immunol 2003;3:269–79. 12669018 10.1038/nri1052

[24] Sportès C , Babb RR, Krumlauf MC, Hakim FT, Steinberg SM, Chow CK, . Phase I study of recombinant human interleukin-7 administration in subjects with refractory malignancy. Clin Cancer Res 2010;16:727–35. 20068111 10.1158/1078-0432.CCR-09-1303 PMC2808195

[25] Daix T , Mathonnet A, Brakenridge S, Dequin P-F, Mira J-P, Berbille F, . Intravenously administered interleukin-7 to reverse lymphopenia in patients with septic shock: a double-blind, randomized, placebo-controlled trial. Ann Intensive Care 2023;13:17. 36906875 10.1186/s13613-023-01109-w PMC10008152

[26] Rosenberg SA , Sportès C, Ahmadzadeh M, Fry TJ, Ngo LT, Schwarz SL, . IL-7 administration to humans leads to expansion of CD8^+^ and CD4^+^ cells but a relative decrease of CD4^+^ T-regulatory cells. J Immunother 2006;29:313–9. 16699374 10.1097/01.cji.0000210386.55951.c2 PMC1473976

[27] Nam HJ , Song M-Y, Choi D-H, Yang S-H, Jin H-T, Sung Y-C. Marked enhancement of antigen-specific T-cell responses by IL-7-fused nonlytic, but not lytic, Fc as a genetic adjuvant. Eur J Immunol 2010;40:351–8. 19950168 10.1002/eji.200939271

[28] Kim MY , Jayasinghe R, Devenport JM, Ritchey JK, Rettig MP, O’Neal J, . A long-acting interleukin-7, rhIL-7-hyFc, enhances CAR T cell expansion, persistence, and anti-tumor activity. Nat Commun 2022;13:3296. 35697686 10.1038/s41467-022-30860-0 PMC9192727

[29] Lee SW , Choi D, Heo M, Shin E-C, Park SH, Kim SJ, . hIL-7-hyFc, A long-acting IL-7, increased absolute lymphocyte count in healthy subjects. Clin Transl Sci 2020;13:1161–9. 32339447 10.1111/cts.12800 PMC7719369

[30] Golumba-Nagy V , Kuehle J, Hombach AA, Abken H. CD28-ζ CAR T cells resist TGF-β repression through IL-2 signaling, which can Be mimicked by an engineered IL-7 autocrine loop. Mol Ther 2018;26:2218–30. 30055872 10.1016/j.ymthe.2018.07.005 PMC6127517

[31] He C , Zhou Y, Li Z, Farooq MA, Ajmal I, Zhang H, . Co-expression of IL-7 improves nkg2d-based CAR T cell therapy on prostate cancer by enhancing the expansion and inhibiting the apoptosis and exhaustion. Cancers (Basel) 2020;12:1969. 32698361 10.3390/cancers12071969 PMC7409228

[32] Li G , Zhang Q, Han Z, Zhu Y, Shen H, Liu Z, . IL-7 and CCR2b Co-Expression-Mediated enhanced CAR-T survival and infiltration in solid tumors. Front Oncol 2021;11:734593. 34778046 10.3389/fonc.2021.734593 PMC8579717

[33] Pang N , Shi J, Qin L, Chen A, Tang Y, Yang H, . IL-7 and CCL19-secreting CAR-T cell therapy for tumors with positive glypican-3 or mesothelin. J Hematol Oncol 2021;14:118. 34325726 10.1186/s13045-021-01128-9 PMC8323212

[34] Li L , Li Q, Yan ZX, Sheng LS, Fu D, Xu P, . Transgenic expression of IL-7 regulates CAR-T cell metabolism and enhances in vivo persistence against tumor cells. Sci Rep 2022;12:12506. 35869100 10.1038/s41598-022-16616-2 PMC9307822

[35] Swainson L , Verhoeyen E, Cosset F-L, Taylor N. IL-7R alpha gene expression is inversely correlated with cell cycle progression in IL-7-stimulated T lymphocytes. J Immunol 2006;176:6702–8. 16709829 10.4049/jimmunol.176.11.6702

[36] Krawczyk E , Zolov SN, Huang K, Bonifant CL. T-Cell activity against AML improved by dual-targeted T cells stimulated through T-cell and IL7 receptors. Cancer Immunol Res 2019;7:683–92. 30782669 10.1158/2326-6066.CIR-18-0748 PMC8186236

[37] Shum T , Omer B, Tashiro H, Kruse RL, Wagner DL, Parikh K, . Constitutive signaling from an engineered IL7 receptor promotes durable tumor elimination by tumor-redirected T cells. Cancer Discov 2017;7:1238–47. 28830878 10.1158/2159-8290.CD-17-0538 PMC5669830

[38] Du X , Ho M, Pastan I. New immunotoxins targeting CD123, a stem cell antigen on acute myeloid leukemia cells. J Immunother 2007;30:607–13. 17667524 10.1097/CJI.0b013e318053ed8e

[39] Bonifant CL , Szoor A, Torres D, Joseph N, Velasquez MP, Iwahori K, . CD123-Engager T cells as a novel immunotherapeutic for acute myeloid leukemia. Mol Ther 2016;24:1615–26. 27401038 10.1038/mt.2016.116 PMC5113097

[40] Zolov SN , Rietberg SP, Bonifant CL. Programmed cell death protein 1 activation preferentially inhibits CD28.CAR-T cells. Cytotherapy 2018;20:1259–66. 30309710 10.1016/j.jcyt.2018.07.005

[41] Vera J , Savoldo B, Vigouroux S, Biagi E, Pule M, Rossig C, . T lymphocytes redirected against the kappa light chain of human immunoglobulin efficiently kill mature B lymphocyte-derived malignant cells. Blood 2006;108:3890–7. 16926291 10.1182/blood-2006-04-017061 PMC1895462

[42] Challis RC , Ravindra Kumar S, Chan KY, Challis C, Beadle K, Jang MJ, . Systemic AAV vectors for widespread and targeted gene delivery in rodents. Nat Protoc 2019;14:379–414. 30626963 10.1038/s41596-018-0097-3 PMC13333184

[43] Christodoulou I , Ho WJ, Marple A, Ravich JW, Tam A, Rahnama R, . Engineering CAR-NK cells to secrete IL-15 sustains their anti-AML functionality but is associated with systemic toxicities. J Immunother Cancer 2021;9:e003894. 34896980 10.1136/jitc-2021-003894 PMC8655609

[44] Kunz A , Gern U, Schmitt A, Neuber B, Wang L, Hückelhoven-Krauss A, . Optimized assessment of qPCR-based vector copy numbers as a safety parameter for GMP-grade CAR T cells and monitoring of frequency in patients. Mol Ther Methods Clin Dev 2020;17:448–54. 32201711 10.1016/j.omtm.2020.02.003 PMC7078460

[45] Hughes CS , Moggridge S, Müller T, Sorensen PH, Morin GB, Krijgsveld J. Single-pot, solid-phase-enhanced sample preparation for proteomics experiments. Nat Protoc 2019;14:68–85. 30464214 10.1038/s41596-018-0082-x

[46] Wang Y , Yang F, Gritsenko MA, Wang Y, Clauss T, Liu T, . Reversed-phase chromatography with multiple fraction concatenation strategy for proteome profiling of human MCF10A cells. Proteomics 2011;11:2019–26. 21500348 10.1002/pmic.201000722 PMC3120047

[47] Herbrich SM , Cole RN, West KP Jr, Schulze K, Yager JD, Groopman JD, . Statistical inference from multiple iTRAQ experiments without using common reference standards. J Proteome Res 2013;12:594–604. 23270375 10.1021/pr300624g PMC4223774

[48] Tyanova S , Temu T, Cox J. The MaxQuant computational platform for mass spectrometry-based shotgun proteomics. Nat Protoc 2016;11:2301–19. 27809316 10.1038/nprot.2016.136

[49] Tyanova S , Temu T, Sinitcyn P, Carlson A, Hein MY, Geiger T, . The Perseus computational platform for comprehensive analysis of (prote)omics data. Nat Methods 2016;13:731–40. 27348712 10.1038/nmeth.3901

[50] Aitchison J . The statistical analysis of compositional data. J R Stat Soc 1982;44:139–60.

[51] van den Boogaart KG , Tolosana-Delgado R, Bren M. compositions: compositional data analysis. R package version 2.0-62023 2024.

[52] Keppler A , Gendreizig S, Gronemeyer T, Pick H, Vogel H, Johnsson K. A general method for the covalent labeling of fusion proteins with small molecules in vivo. Nat Biotechnol 2003;21:86–9. 12469133 10.1038/nbt765

[53] Keppler A , Pick H, Arrivoli C, Vogel H, Johnsson K. Labeling of fusion proteins with synthetic fluorophores in live cells. Proc Natl Acad Sci U S A 2004;101:9955–9. 15226507 10.1073/pnas.0401923101 PMC454197

[54] Los GV , Encell LP, McDougall MG, Hartzell DD, Karassina N, Zimprich C, . HaloTag: a novel protein labeling technology for cell imaging and protein analysis. ACS Chem Biol 2008;3:373–82. 18533659 10.1021/cb800025k

[55] Guedan S , Calderon H, Posey AD Jr, Maus MV. Engineering and design of chimeric antigen receptors. Mol Ther Methods Clin Dev 2019;12:145–56. 30666307 10.1016/j.omtm.2018.12.009 PMC6330382

[56] Maher J , Brentjens RJ, Gunset G, Rivière I, Sadelain M. Human T-lymphocyte cytotoxicity and proliferation directed by a single chimeric TCRzeta /CD28 receptor. Nat Biotechnol 2002;20:70–5. 11753365 10.1038/nbt0102-70

[57] Bridgeman JS , Ladell K, Sheard VE, Miners K, Hawkins RE, Price DA, . CD3ζ-based chimeric antigen receptors mediate T cell activation via cis- and trans-signalling mechanisms: implications for optimization of receptor structure for adoptive cell therapy. Clin Exp Immunol 2014;175:258–67. 24116999 10.1111/cei.12216 PMC3892417

[58] Guedan S , Posey AD Jr., Shaw C, Wing A, Da T, Patel PR, . Enhancing CAR T cell persistence through ICOS and 4-1BB costimulation. JCI Insight 2018;3:e96976. 29321369 10.1172/jci.insight.96976 PMC5821198

[59] Wan Z , Shao X, Ji X, Dong L, Wei J, Xiong Z, . Transmembrane domain-mediated Lck association underlies bystander and costimulatory ICOS signaling. Cell Mol Immunol 2020;17:143–52. 30523347 10.1038/s41423-018-0183-z PMC7000777

[60] Davenport AJ , Cross RS, Watson KA, Liao Y, Shi W, Prince HM, . Chimeric antigen receptor T cells form nonclassical and potent immune synapses driving rapid cytotoxicity. Proc Natl Acad Sci U S A 2018;115:E2068–76. 29440406 10.1073/pnas.1716266115 PMC5834689

[61] Sajman J , Yakovian O, Unger Deshet N, Almog S, Horn G, Waks T, . Nanoscale CAR organization at the immune synapse correlates with CAR-T effector functions. Cells 2023;12:2261. 37759484 10.3390/cells12182261 PMC10527520

[62] Vetrie D , Helgason GV, Copland M. The leukaemia stem cell: similarities, differences and clinical prospects in CML and AML. Nat Rev Cancer 2020;20:158–73. 31907378 10.1038/s41568-019-0230-9

[63] Stefanski HE , Eaton A, Baggott C, Rossoff J, Verneris MR, Prabhu S, . Higher doses of tisagenlecleucel are associated with improved outcomes: a report from the pediatric real-world CAR consortium. Blood Adv 2023;7:541–8. 35938863 10.1182/bloodadvances.2022007246 PMC9979765

