# Supplementary material for: Activation of Cell-Intrinsic Signaling in CAR-T Cells via a Chimeric IL7R Domain
Source: Cancer Res Commun. 2024 Sep 9;4(9):2359–73. doi: 10.1158/2767-9764.CRC-24-0286 (PMC11382189; doi:10.1158/2767-9764.CRC-24-0286)
Supplement: Figure S1 — Supplementary Figure 1 [file crc-24-0286_figure_s1_suppsf1.pdf]

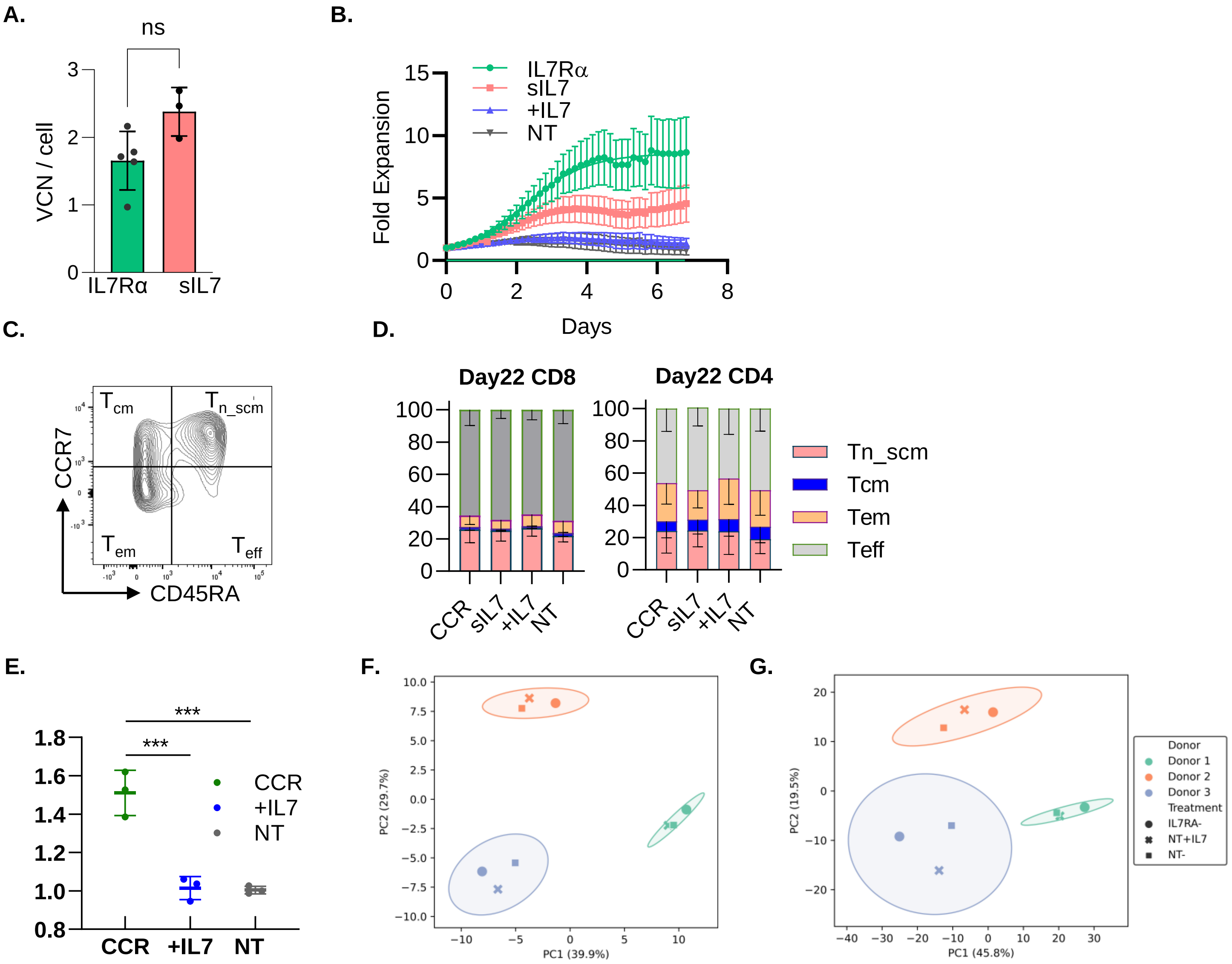

Supplemental Figure 1. Expression of IL7R-CCR or secreted IL7 can stimulate proliferation and activation of engineered T cells independent of exogenous cytokine supplementation. A. Vector Copy Number (VCN) per cell in modified T cells. **B.** IL7R CCR, sIL7, and unmodified T cell cytokine-independent growth measured over 1 week. +IL7 group indicates supplementation during expansion prior to plating. n=4 independent T-cell donors, data presented as fold increase normalized to time 0. **C.** Representative gating strategy to define T-cell subsets based on CCR7 and CD45RA expression. **D.** Immunophenotype of healthy donor PBMCs and transduced T cells on days 22 of *in vitro* culture. **E.** MS Proteomics analysis detects CCR scFv with high fidelity. Raw detected protein abundance was normalized to the median control condition and expressed as mean Log2 fold-change of three donors.(n=3 independent T-cell donors). ***p<0.001. **F.** PCA plots of proteomic analysis at the total protein and **G.** phosphosite level. n=3 independent T-cell donors.
