# Supplementary material for: Activation of Cell-Intrinsic Signaling in CAR-T Cells via a Chimeric IL7R Domain
Source: Cancer Res Commun. 2024 Sep 9;4(9):2359–73. doi: 10.1158/2767-9764.CRC-24-0286 (PMC11382189; doi:10.1158/2767-9764.CRC-24-0286)
Supplement: Figure S2 — Supplementary Figure 2 [file crc-24-0286_figure_s2_suppsf2.pdf]

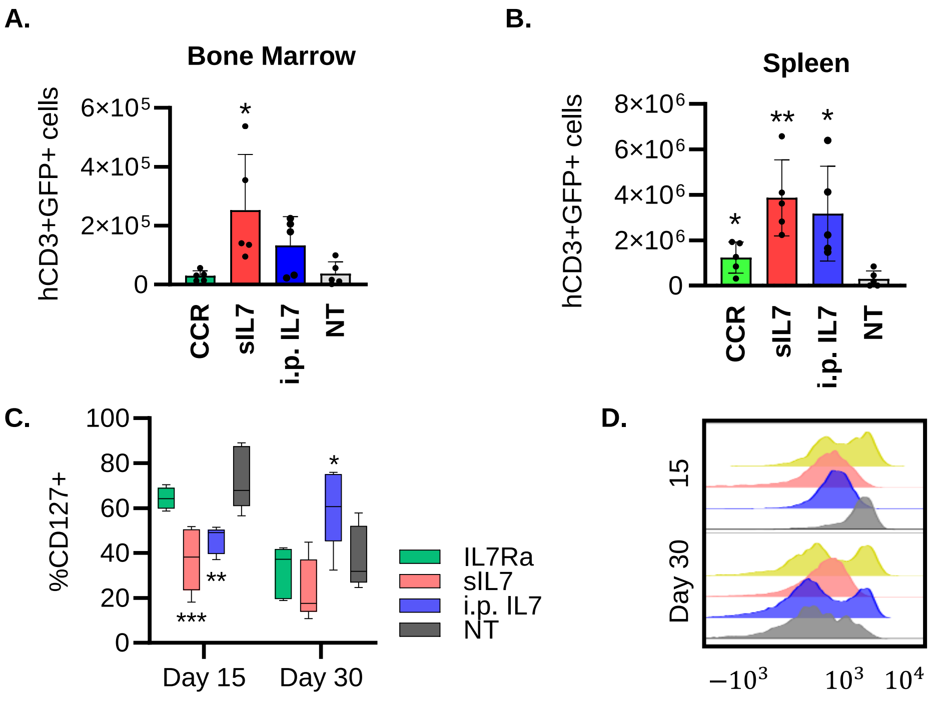


**Supplemental Figure 2. IL7 pathway activated T cells are detected and demonstrate dynamic CD127 expression after adoptive transfer. A**. Number of GFP+ human T-cells in the bone marrow and **B.** spleen of mice upon sacrifice on day 38. **C.** Expression of the native IL7Rα chain on circulating T cells (%CD127+) of each cohort. **D.** Corresponding histograms of a representative sample per group depicting CD127 percent expression on hCD3+GFP+. For all panels: n=5 individual mice per group, comparison to control group (NT). *p<0.05, **p-value<0.01, ***p<0.001
