# Supplementary material for: Activation of Cell-Intrinsic Signaling in CAR-T Cells via a Chimeric IL7R Domain
Source: Cancer Res Commun. 2024 Sep 9;4(9):2359–73. doi: 10.1158/2767-9764.CRC-24-0286 (PMC11382189; doi:10.1158/2767-9764.CRC-24-0286)
Supplement: Figure S3 — Supplementary Figure 3 [file crc-24-0286_figure_s3_suppsf3.pdf]

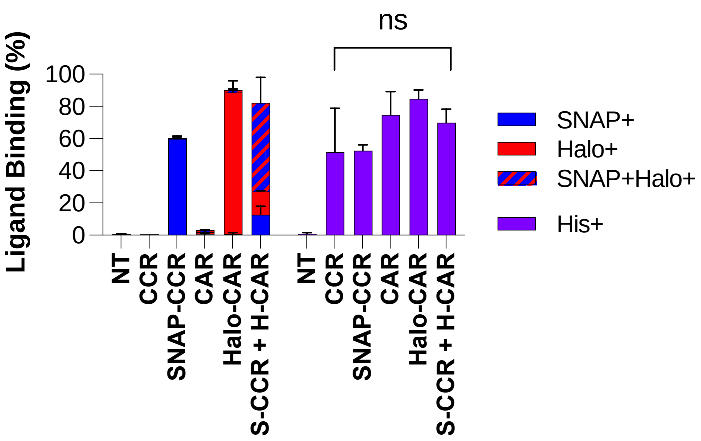

Supplemental Figure 3. Addition of chemical Halo and SNAP tags do not affect CCR/CAR expression or CD123 binding. Transduction efficiency measured using Halo- and SNAP-ligands versus coating of cells with recombinant human CD123-His followed by anti-His staining.
