# Supplementary material for: Activation of Cell-Intrinsic Signaling in CAR-T Cells via a Chimeric IL7R Domain
Source: Cancer Res Commun. 2024 Sep 9;4(9):2359–73. doi: 10.1158/2767-9764.CRC-24-0286 (PMC11382189; doi:10.1158/2767-9764.CRC-24-0286)
Supplement: Figure S4 — Supplementary Figure 4 [file crc-24-0286_figure_s4_suppsf4.pdf]

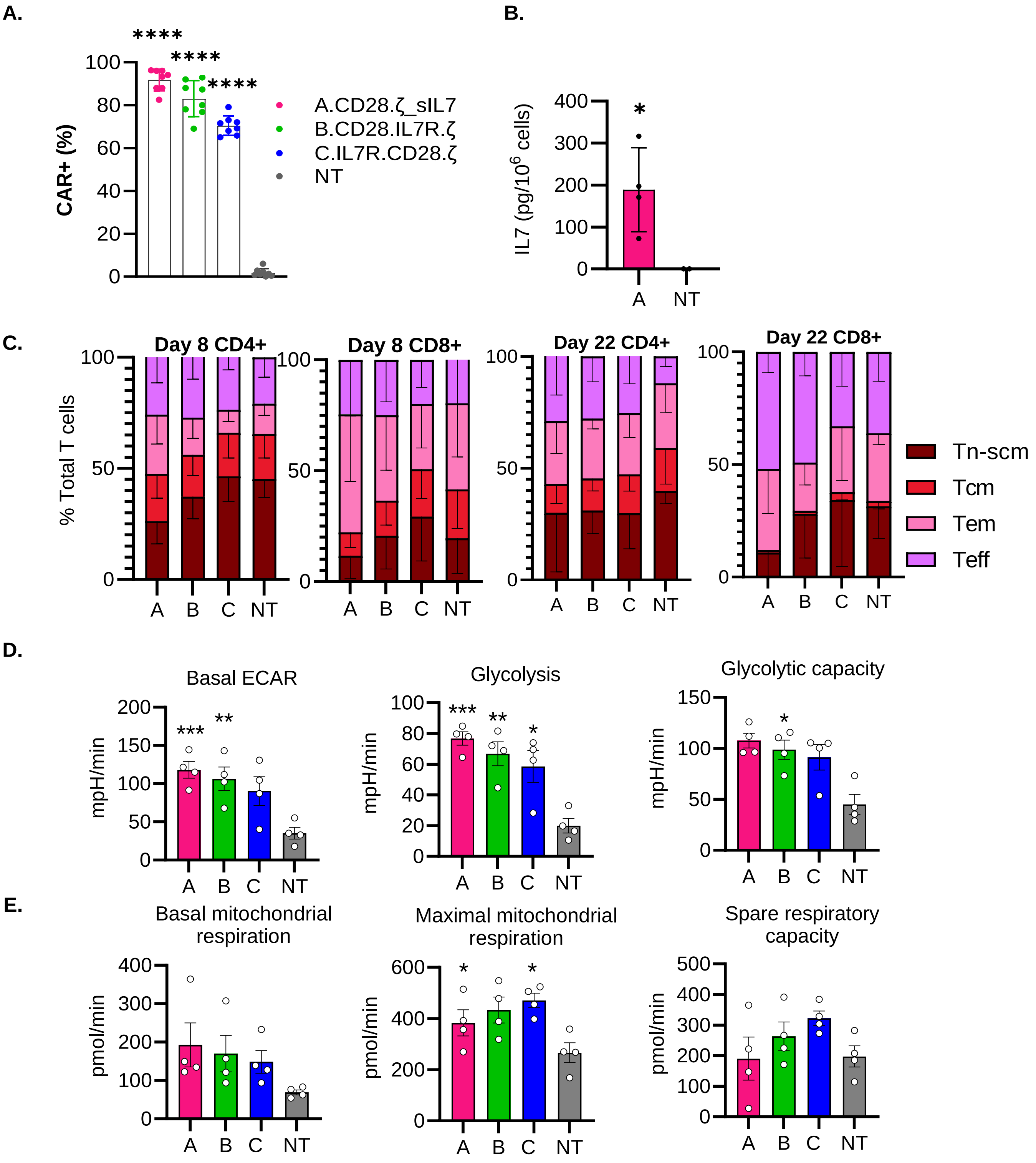

Supplemental Figure 4. IL7R functional activation alters the phenotype of CAR-T cells. A. Transduction efficiency of constructs measured by percent CAR+ cells on flow cytometry **B.** IL7 concentration in the supernatant of IL7-secreting T cells, measured by ELISA. **C.** Immunophenotype of the cells on day 8 and day 22 of *in vitro* culture. For B. and C., n=4-6 individual T-cell donors. *p<0.05, ***p<0.0001. **D.** Extracellular acidification rate of T cells measured by extracellular flux assays after 24-hour stimulation on plate-bound recombinant antigen (rhCD123) in cytokine-free media **E.** Oxygen consumption rate of T cells measured by extracellular flux assay after 24-hour stimulation on rhCD123 in cytokine-free media. D. and E. depict n=4 individual donors tested in five replicates per assay. Seahorse curves represent data normalized to control T cells for each donor. *p<0.05, **p<0.01, ***p<0.001.
