# Supplementary material for: Activation of Cell-Intrinsic Signaling in CAR-T Cells via a Chimeric IL7R Domain
Source: Cancer Res Commun. 2024 Sep 9;4(9):2359–73. doi: 10.1158/2767-9764.CRC-24-0286 (PMC11382189; doi:10.1158/2767-9764.CRC-24-0286)
Supplement: Figure S5 — Supplementary Figure 5 [file crc-24-0286_figure_s5_suppsf5.pdf]

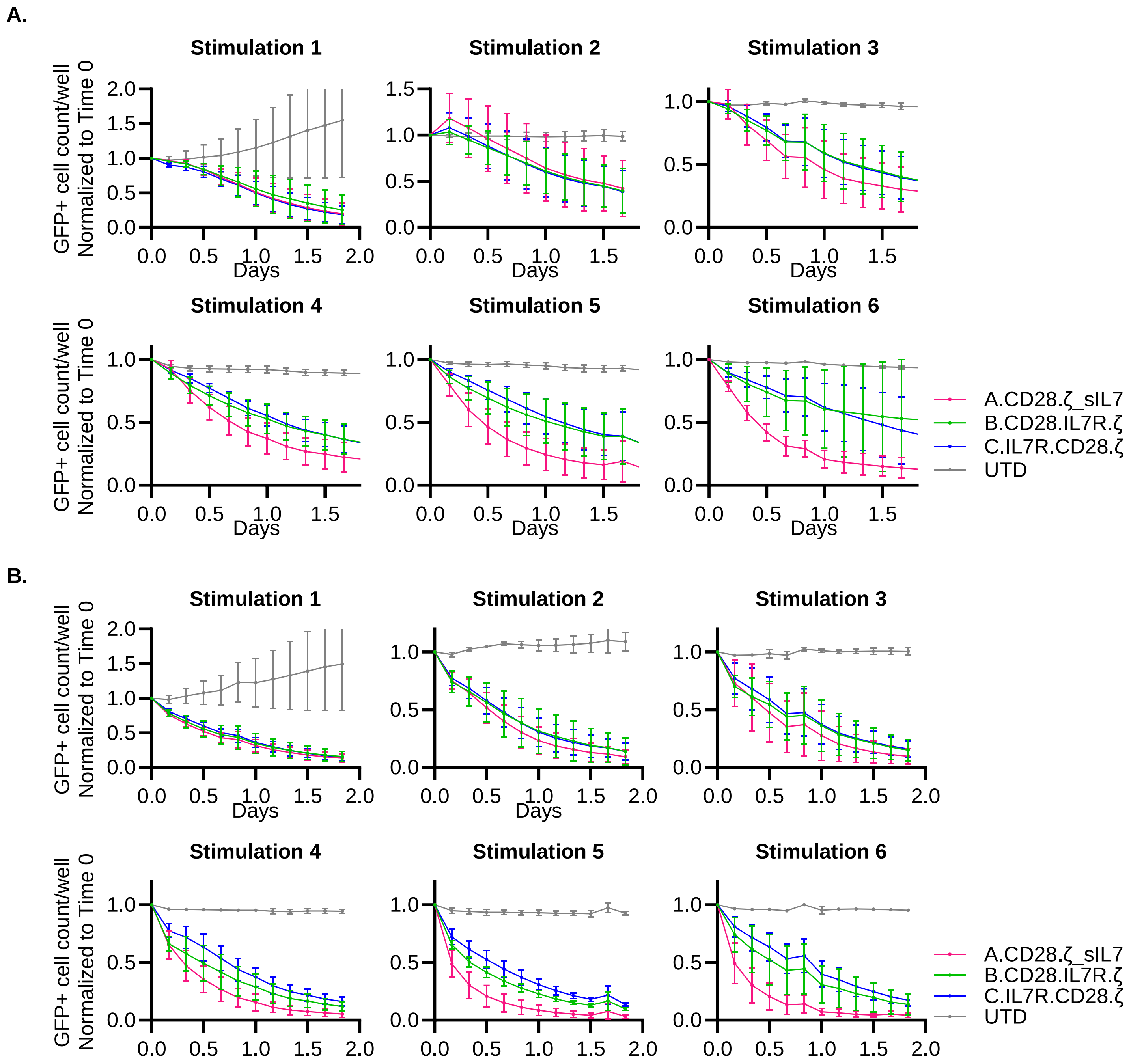

Supplemental Figure 5. IL7R pathway activation does not inhibit long-term CAR-T cell specific anti-tumor cytotoxicity. A. Individual cytotoxicity during 48 hr serial stimulations of indicated T cell populations with MV-4-11 and B. Molm-13 when plated at an initial 1:1 Effector:Target ratio. n=4 unique T-cell donors.
