# Supplementary material for: Activation of Cell-Intrinsic Signaling in CAR-T Cells via a Chimeric IL7R Domain
Source: Cancer Res Commun. 2024 Sep 9;4(9):2359–73. doi: 10.1158/2767-9764.CRC-24-0286 (PMC11382189; doi:10.1158/2767-9764.CRC-24-0286)
Supplement: Supplementary Tables — Supplemental Table 1 and 2 [file crc-24-0286_supplementary_tables_suppst1-st2.pdf]

NLS-eGFP	MSSDDEATADSQHSTPPKKKRKVEDPMVSKGEELFTGVVPILVELDGDVNGHKFSVSGEGEGDATYGKLTLKFICTTGKLPVPWPTLVTTLTYGVQCFSRYPDHMKQHDFFKSAMPEGYVQERTIFFKDDGNYKTRAEVKFEGDTLVNRIELKGIDFKEDGNILGHKLEYNYNSHNVYIMADKQKNGIKVNFKIRHNIEDGSVQLADHYQQNTPIGDGPVLLPDNHYLSTQSALSKDPNEKRDHMVLLEFVTAAGITLGMDELYK
sIL7	MATTFHVSFRYIFGLPPLILVLLPVASSDCDIEGKDGKQYESVLMVSIDQLLDSMKEIGSNCLNNEFNFFKRHICDANKEGMFLFRAARKLRQFLKMNSTGDFDLHLLKVSEGTTILLNCTGQVKGRKPAALGEAQPTKSLEENKSLKEQKKLNDLCFLKRLLQEIKTCWNKILMGTKEH
IL7Rα Transmembrane	PILLTISILSFFSVALLVILAVLW
IL7Rα Intracellular	KKRIKPIVWPSLPDHKKTLEHLCKKPRKNLNVSFNPESFLDCQIHRVDDIQARDEVEGFLQDTFPQQLEESEKQRLGGDVQSPNCPSEDVVITPESFGRDSSLTCLAGNVSACDAPILSSSRSLDCRESGKNGPHVYQDLLLSLGTTNSTLPPPFSLQSGILTLNPVAQGQPILTSLGSNQEEAYVTMSSFYQNQ

Supplemental Table 1. Amino acid sequences of employed transgenic elements.

Target	Clone	Fluorophore	Source	Catalog Number
T cell activation				
**CD3**	OKT3		Miltenyi	130-093-387
**CD28**	CD28.2		Fisher	555725
Immunophenotyping				
**Viability Stain 780**			BD Biosciences	65388
**His**	J095G46	APC	BioLegend	362605
**CD4**	RPA-T4	BUV805	BD Biosciences	742000
**CD8**	RPA-T8	BV421	BD Biosciences	562428
**CD45RA**	HI100	BV510	BD Biosciences	563031
**CD197 (CCR7)**	G043H7	BV605	BioLegend	353224
**CD95**	DX2	BV786	BD Biosciences	740991
**CD127**	HIL-7R-M21	PE	BD Biosciences	557938
**CD3**	UCHT1	BV605	BD Biosciences	742623
**CD33**	P67.6	BV421	BD Biosciences	744761
**CD45**	HI30	APC	BioLegend	304012
Intracellular stain				
**pSTAT5 (pY694)**	47/Stat5(pY694)	AlexaFluor647	BD Biosciences	612599
**Western blot**				
**GAPDH**	6C5		Invitrogen	AM4300
**STAT5**	D2O6Y		Cell Signaling	94205S
**pSTAT5 (pY694)**	D47E7		Cell Signaling	4322S

Supplemental Table 2. Comprehensive list of antibodies used in study
